# The design of functional proteins using tensorized energy calculations

**DOI:** 10.1016/j.crmeth.2023.100560

**Published:** 2023-08-15

**Authors:** Kateryna Maksymenko, Andreas Maurer, Narges Aghaallaei, Caroline Barry, Natalia Borbarán-Bravo, Timo Ullrich, Tjeerd M.H. Dijkstra, Birte Hernandez Alvarez, Patrick Müller, Andrei N. Lupas, Julia Skokowa, Mohammad ElGamacy

**Affiliations:** 1 Department of Protein Evolution, Max Planck Institute for Biology, 72076 Tübingen, Germany; 2 Friedrich Miescher Laboratory of the Max Planck Society, 72076 Tübingen, Germany; 3 Werner Siemens Imaging Center, Department of Preclinical Imaging and Radiopharmacy, Eberhard Karls University, 72076 Tübingen, Germany; 4 Cluster of Excellence iFIT (EXC 2180) “Image Guided and Functionally Instructed Tumor Therapies,” Eberhard Karls University, 72076 Tübingen, Germany; 5 Division of Translational Oncology, University Hospital Tübingen, 72076 Tübingen, Germany; 6 Krieger School of Arts and Sciences, Johns Hopkins University, Washington, DC 20036, USA; 7 Department for Women’s Health, University Hospital Tübingen, 72076 Tübingen, Germany; 8 Translational Bioinformatics, University Hospital Tübingen, 72072 Tübingen, Germany

**Keywords:** protein design, energy calculation, discrete rotamer sampling, EGFR inhibitor, copper binder

## Abstract

In protein design, the energy associated with a huge number of sequence-conformer perturbations has to be routinely estimated. Hence, enhancing the throughput and accuracy of these energy calculations can profoundly improve design success rates and enable tackling more complex design problems. In this work, we explore the possibility of tensorizing the energy calculations and apply them in a protein design framework. We use this framework to design enhanced proteins with anti-cancer and radio-tracing functions. Particularly, we designed multispecific binders against ligands of the epidermal growth factor receptor (EGFR), where the tested design could inhibit EGFR activity *in vitro* and *in vivo*. We also used this method to design high-affinity Cu^2+^ binders that were stable in serum and could be readily loaded with copper-64 radionuclide. The resulting molecules show superior functional properties for their respective applications and demonstrate the generalizable potential of the described protein design approach.

## Highlights

• Tensorized energy calculations accelerate protein design
• A rotamer library can cover residues and ligands not present in structure databases
• The design framework does not rely on any training data
• This framework is applicable to diverse protein design problems

## Motivation

Calculating the interaction energies between atoms is a central process in biomolecular simulations. Traditionally, these calculations are performed exhaustively for each atom pair, which constitutes the computational bottleneck. In this study, we introduce a framework that instead represents the dense atomic interaction fields as three-dimensional projections. These projections can condense energy evaluations into a single matrix operation, greatly simplifying the computational load. We apply this framework to the complex protein design problem in order to identify a low-energy amino acid sequence for a target structure.

## Graphical abstract

**Figure:**
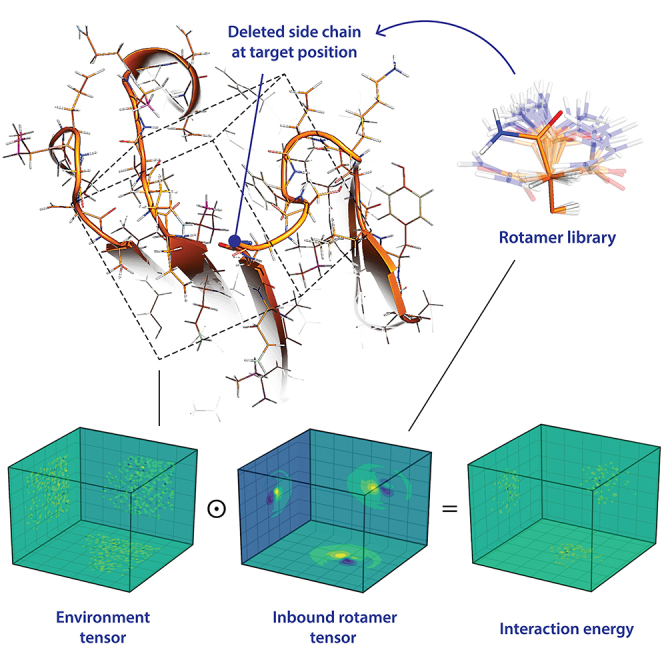

## Teaser

Maksymenko et al. present an approach for protein design that accelerates interaction energy calculations, generates a rotamer library relying only on force-field parameters, and offers a training-free scoring function. The authors apply this framework to design proteins with EGFR-inhibiting and radio-tracing functions.

## Introduction

Protein design processes search for sequences to fill up a given target structure while minimizing the free energy of this defined configuration. Under the layout of fixed-backbone design, amino acid side chains and conformations are sampled at the designable positions and scored for their energy within their local environment. Thus, protein design simulations typically sample a large number of sequence-conformer combinations even for a small number of designable positions. Moreover, the computational load increases steeply with the difficulty of the specific design problem.^1^ This demands scoring functions to be sufficiently fast to cover large sequence sub-spaces that contain viable solutions.^2^ The inherent trade-off between the scoring speed and the accuracy has led to the broad utility of fast energy functions and trained or knowledge-based models. Previous efforts include the use of knowledge-based scoring terms,^3^ coarse-grained representation,^4^ geometric filters,^5^ and directly^6^ or indirectly^7^ learned sequence-structure relationships.

**Figure 1:**
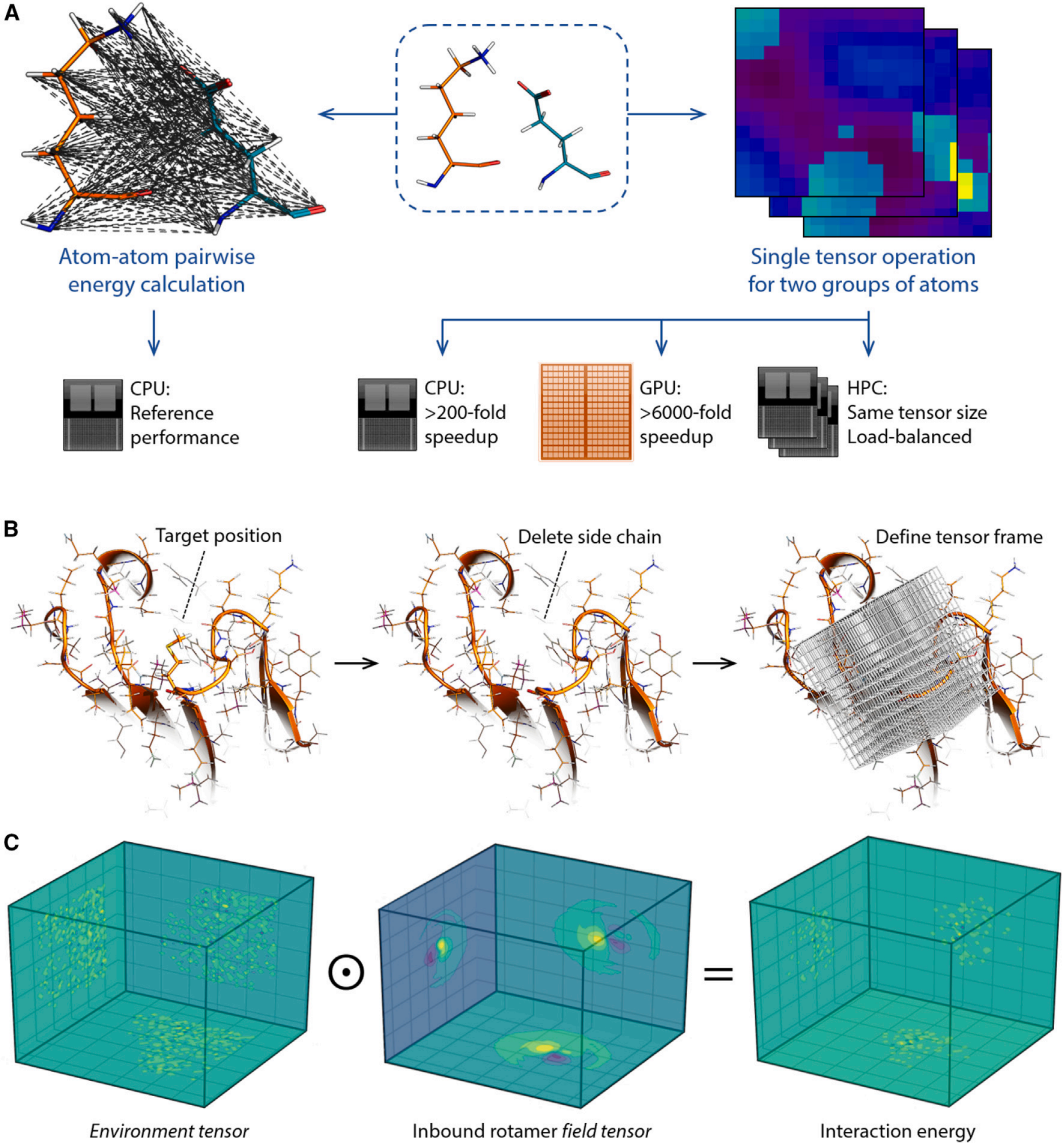
The concept of the tensorized design framework (A) The non-bonded interactions between an amino acid and its molecular environment (e.g., another proximal amino acid) entail the calculation of all atom-atom pairwise potential energies within a distance cutoff. The intensive execution of a large number of distance- and energy-evaluation instructions, as well as memory handling processes, slows the overall performance. Thus, formulating the energy evaluation problem between two groups of atoms as a single tensor operation not only speeds up the scoring on conventional processors, but also renders the calculation highly compatible with stream processors. Moreover, the constant dimensions of the used tensors enable ideal load balancing on high-performance computers. The performance enhancement figures were calculated for the Lennard-Jones potential function, a cubic tensor representation of 22 Å side and 0.5 × 0.5 × 0.5 Å voxels. The computing operations are performed as in (B) and (C). (B) Once a mutable or repackable target residue is defined, the input structure is transformed to the frame of reference with respect to the residue’s backbone coordinates. The side-chain atoms of the target residue are then deleted, leaving behind the “environment” atoms. (C) Proxy values for the atoms’ positions, partial charges (illustrated here as plane projections; yellow, negative; blue, positive) or their surface solvation energies are projected onto the voxels of a constructed tensor. The rotamer library comprises the more expensive, pre-computed smooth interaction fields (i.e., field tensors; example shown for an asparagine side chain), which through a single element-wise multiplication with the environment tensor yields the spatially resolved energy in a “two-body” format.

In this work, we explore the feasibility of tensorizing energy calculations for molecular mechanics applications and, particularly, evaluate its usefulness for protein design simulations. In protein design, the evaluation of the energy associated with non-bonded interactions represents the computational bottleneck. We seek to demonstrate the accessible performance gains from reformulating the non-bonded energy terms (i.e., the Lennard-Jones [LJ], electrostatic, and solvation energy terms) to best suit the computing hardware architecture. Specifically, conducting energy calculations as large-matrix (or tensor) operations enables substantial efficiency gains on both conventional central processing units (CPUs) and massively parallel processing hardware (Figure 1A). Here we use an energy function that is readily derived from established, self-consistent force fields. This obviates the need for empirically optimizing a scoring function against one or more training datasets and thus avoids overfitting and training bias. Our approach also attributes inaccuracies directly to the force-field parameters, allowing improvements to be more systematic. Finally, as tensorization increases the throughput of evaluating sequence:conformer combinations, it raises the probability of finding lower-energy solutions, which can improve the experimental success rate.

As an overview, our design workflow starts by pre-computing a discrete rotamer library from molecular dynamics (MD) simulations of isolated amino acids. These simulations are run under the same force field from which the scoring function terms are derived. The resulting conformational pool of each amino acid is then partitioned into clusters with a constant number of rotamers. Each rotamer in the database is dually represented by its atomic coordinates and by its interaction fields projections (field tensors). To evaluate the interaction energy between an inbound rotamer and a host structure, the existing side chain at the designable position is removed (Figure 1B), and the surrounding environment is projected as a three-dimensional histogram of the constituting atoms (environment tensor). The element-wise multiplication of a rotamer’s field tensor and a structure’s environment tensor yields the interaction energy between them (Figure 1C).

We use this framework for designing two different classes of proteins. The first class aims at inhibiting soluble growth factors, particularly, the epidermal growth factor (EGF)-like ligands. Multispecific quenchers of the several soluble ligands of the EGF receptor (EGFR) are needed for broad inhibition of EGFR signaling and can serve as a new therapeutic modality for several types of cancer.^8^ In this work, we redesigned a minimal receptor domain to maximally stabilize it in its ligand-bound form. This yielded small, single-domain binders (18 kDa) that are biophysically and functionally superior to the natural template. The designs showed multispecific binding to their target growth factors and potently inhibited EGFR signaling *in vitro* and *in vivo*. Our second objective is to develop a new class of protein-based radiotracers for use as genetically encodable molecules for positron-emission tomography (PET) imaging. Using our framework, we could redesign a natural copper-storage protein into a developable form as a diagnostic protein tag. Two designs were monomeric and showed superior stability and high production yield, in contrast to the starting template. These designs were capable of binding Cu^2+^ with high affinity and low off rates, and, importantly, were sufficiently stable for hours in serum *in vitro*, highlighting their translational potential.

## Results and discussion

### Rotamer library construction

Typically, discrete rotamer libraries are constructed from amino acid conformations pooled from known structures. As the structural databases grew in size, more stringent inclusion criteria were imposed, greatly improving the quality of the available libraries.^9^ Nonetheless, PDB-based rotamer libraries still provide a sparse coverage of the rotameric space and entrain undesirable factors pertinent to protein structure determination, such as cryogenic measurement condition or ensemble averaging. Thus, we use MD to achieve more extensive sampling of the conformational tendencies of amino acids in folded proteins.^10^ Furthermore, MD simulations of isolated amino acids can cover a broader conformational distribution that is unbiased by the choice of input protein structures. Such conformational distributions more faithfully reproduce the tendencies of the random coil state and represent a reference energy distribution prior to any folding event.^11^ We have therefore chosen to build the rotamer library using MD simulations of capped amino acids (i.e., Ac-X-NHMe).^12^ The internal energies related to backbone conformational preferences were derived from the occupancy of backbone bins of the dihedral space (i.e., (φ,ψ) angles). The side-chain conformational preferences were nonetheless mapped in the Cartesian space by means of root-mean-square deviation (RMSD) clustering after alignment to a (C^β^-C^α^-N) frame of reference. This was sought to obtain a constant number of rotamer clusters for every (φ,ψ) bin, regardless of the number of atoms or conformational tendencies of the amino acid. Each cluster would be represented by a single rotamer in the final library, where the relative energy of the rotamer relates to its respective cluster size (STAR Methods).

This approach brings several advantages. First, the internal energies derived from the conformational preferences of amino acids are consistent with the other non-bonded energy terms used in design calculations, as both rely on the same force field. Second, the generated rotamers can implicitly encode the dynamic influences on bond angles, bond stretching, and improper dihedrals. These subtle deformations are generally dismissed in other rotamer libraries, but have been shown to significantly impact the energy gap between native and non-native states of a protein.^13^ Third, this approach offers great versatility regarding the energy function of choice. Whereas here we used the CHARMM force field^14^ for both the rotamer library creation and the design energy function, more complex potentials (e.g., polarizable force fields) can be deployed. The library can be readily extended to cover rotameric distributions in different pH or sequence (e.g., tri- or pentapeptide) contexts and can generate on-demand rotamers for non-standard amino acids or ligands in a consistent way.^15^ Fourth, the long MD trajectories effectively sample a broader space compared with what is traditionally obtained from the PDB-derived rotamer libraries^16^ (Figure S1A). Furthermore, by raising the temperature of the isolated amino acid MD simulations, broader coverage of otherwise poorly sampled (φ,ψ) regions can be covered more effectively than in PDB-based rotamer libraries (Figure S1B). The former can be particularly successful in better accessing rare “linchpin” rotamers reported to constitute sampling bottlenecks.^17^ Last, the extraction of a constant number of rotamers plays an important role downstream of the algorithm, as it dictates a defined rotameric sampling granularity. It also guarantees a uniform load balancing during the design calculations, particularly in parallelized implementations.

### Tensorized molecular interaction fields, energies, and mechanics

Our framework combines two principles to maximize the computational efficiency of energy calculations. The first principle is to pre-compute and store most of the information needed for energy calculation. The second principle is to deploy a tensorized form of the energy functions to better fit a single-instruction, multiple data processing paradigm, a hallmark of modern computing technology (Figure 1A).

In this framework, the scoring problem is simplified into a multiatom, two-body problem, where the 1^st^ body represents the multiatom chemical environment surrounding the side chain at the designable position, wherein the atoms of this side chain are absent. The 2^nd^ body represents the inbound side-chain rotamer, aligned to the same frame of reference. The information on this two-body interaction is encoded in an asymmetric fashion in which the 1^st^ body only encodes the three-dimensional occupancy of its atomic positions and charges, while the 2^nd^ body encodes the net, real-valued energy field around all of its respective atoms (Figure 1C). The computationally expensive step is the projection of energy fields, which in this case is restricted to the 2^nd^ body (i.e., the rotamer) and is hence pre-computed once and stored in a look-up table. This restricts the run-time computing load to simply mapping the 1^st^-body three-dimensional occupancy, substantially reducing the run time needed at every designable position. Such a representation benefits from further speed-up when implemented in a tensorized fashion. In this manner, the scalar-valued interaction energy between the two bodies is obtained by the sum of the element-wise product of the two tensors (representing environment atoms and the rotamer energy field). This format is ideally suited for evaluating both the LJ and the electrostatic potentials, albeit at the cost of assuming symmetric LJ parameters for the interacting atom pairs, according to the atom’s respective encoding in the 2^nd^ body tensor. Nonetheless, an atom-type rescaling of atoms in the 1^st^ body can be used to correct for this in the future. Applying the tensorization framework would differ, however, in the case of encoding a surface area-based solvation potential. In the latter situation, the 1^st^ body tensor has to fully describe the environment’s surface solvation energy field. Hence, the solvation energy per unit surface area will be normalized by the number of voxels representing the atomic surface (STAR Methods). Such a tensor is pre-computed for the 2^nd^ body (i.e., the inbound side-chain rotamer) and is computed on the fly for the 1^st^ body as well as for the combined two bodies (STAR Methods). This renders the solvation term the relatively more expensive energy term to compute.

To assess the accuracy of this energy function retrospectively, we predict the energy change associated with single-point mutations. To avoid the confounding effects of combinatorial repacking and iterative energy minimization, the energy values were evaluated without any combinatorial side-chain optimization, but by finding the lowest-energy rotamer at the designated position only (STAR Methods). We used the dataset of mutants of the β1 domain of streptococcal protein G, constituting the largest thermodynamic stability dataset collected in a single experimental setup to date.^18^ This dataset covers most of the single-point mutagenesis landscape of the Gβ1 protein and represents a broad range of burial and secondary structure contexts. In this setup, ΔΔG values obtained by the tensorized potential (herein referred to as the Damietta potential) showed a better correlation ($R=0.46,p=7.3\times 10^{-34}$) compared with the reported Rosetta score^18^ ($R=0.36,p=2.6\times 10^{-21}$) (Figure S2), where both methods were used without minimization. By testing our potential against other large datasets generated from proteolytic stability assays,^19^ we obtained ΔΔG correlation coefficients ranging between 0.26 and 0.41. These datasets comprise diverse folds of the N-terminal domain of the phage 434 repressor (1,046 mutants; $R=0.41,p=1.1\times 10^{-43}$), the SH3 domain of human obscurin (1,097 mutants, $R=0.26,p=2.1\times 10^{-18}$), the N-terminal domain of ribosomal protein 493 L9 (725 mutants, $R=0.35,p=4.9\times 10^{-22}$), and r11_829_TrROS protein designed by trRosetta (833 mutants, $R=0.31,p=1.7\times 10^{-20}$) (Figure S2). We also sought to evaluate the native side-chain conformer recovery for a dataset of proteins with available X-ray and NMR structures.^6^ The overall recovery rates obtained by the Damietta potential were around 70% for χ_1_ and 50% for χ_1&2_ (Figure S3A). As expected, buried residues had a higher prediction accuracy (∼90% for χ_1_ and 65%–85% for χ_1&2_; Figure S3B), given the constraining chemical environment around the amino acids at the protein core. We further used the same dataset of X-ray structures to evaluate native sequence recovery, where the results showed very low recovery rates compared with other design methods (Figure S3C). This can be attributed to the fact that our potential was not trained to maximize sequence nativeness, which is not necessarily a proxy of sequence optimality.

### Combinatorial design by decision tree swarm

The highly dimensional nature of combinatorial design of more than a few amino acid positions severely limits the usefulness of exact sampling algorithms in finding global minimum solutions within reasonable computing time frames. Nonetheless, despite the non-additive effects of correlated mutations, favorable mutations generally tend to cluster closely in the sequence space.^20^ Thus, a swarm of greedy samplers traversing several sequence optimization paths simultaneously can generally reduce entrapment within local minima and lead to near-optimal solutions. This is clearly demonstrated by the success of stochastic design algorithms.^21^ In order to enable the exploration of a sizable number of mutations, we developed a combinatorial sampling strategy that searches for successive minima by spanning a few semi-independent search paths. This strategy builds on the power of parallel, loosely communicating conformational samplers that assume a globally smooth, but locally rugged, landscape as was demonstrated with the SARS^22^ and FLAPS^23^ algorithms. One way of implementing this approach in a design context is through a “few-to-many, many-to-few” scheme, whereby designable amino acid positions are arranged as depth levels within a decision tree, and nodes at each level represent the mutational decisions. As branching represents the expansion to many mutant combinations, a ranking-and-trimming step that keeps only a few lowest-energy designs is imposed between the layers of the tree (Figure S4). This alternation between the many combinations and the few best intermediary decoys guarantees the traversal of a pre-set number of branches (n_paths) down the tree depth at any given level. This scheme restrains the combinational load complexity and enables several parallelization schemes. Measuring the overall performance of the algorithm against varying design loads shows the algorithm to greatly simplify the sampling complexity, while enabling an arbitrary level of parallel sampling through the number of paths (n_paths). These paths not only generate diverse output from a single run, but also keep track of several local minima, which improves the overall minimization outcome when the sampling complexity grows. Particularly, under the same sampling complexity, minimizing across a larger number of paths leads to lower energy decoys (Table S1). These results also highlight a rotamer sampling performance in the microsecond range, which, compared with other methods, indicates substantial performance gains (Table S1). We expect further performance optimization to greatly improve these figures in the future.

In most stochastic design algorithms, higher energy mutations can still be accepted at a lower probability in favor of basin hopping and diversity generation. This, however, adds random noise to the already heterogeneous uncertainty of the scoring function (i.e., scoring error). Instead, here we introduce diversity by randomizing the ordering of the designable positions along the decision tree (i.e., across independent design simulation replicas). In this setup, the parallel, deterministic sampling trials are more dispersed across the solution space and their optimization paths can be easily retraced, in comparison with the use of a Metropolis criterion. This “few-to-many-to-few” combinatorial sampler is thus best run through several independent replicas (while randomizing the order of designable positions) with iterated traversals of the same decision tree ($n_{iter}$) in order to improve the search convergence within each simulation replica (Figure S4).

### Design and characterization of EGFR inhibitors

As a proof-of-principle, we applied our framework to create inhibitors of EGFR signaling, a key pathway involved in the survival, proliferation, and dissemination of tumor cells.^24^ EGFR (HER1) is a receptor tyrosine kinase that represents an important target for modulating signal transduction cascades, as it dimerizes upon ligand-induced conformational change.^25^ Approved inhibitors of EGFR signaling are either small-molecule inhibitors of the receptor’s intracellular kinase domain or monoclonal antibodies blocking its ectodomain dimerization.^26^ These are indicated for treating different EGFR-dependent cancers, e.g., colon cancer and epidermoid carcinomas.^27^ Nonetheless, these two inhibition modalities (i.e., tyrosine kinase inhibitors and dimerization-inhibiting monoclonal antibodies) have been shown to be subject to evasion by cancer cells through numerous evolution and resistance mechanisms.^26^ Binders targeting the ligand itself, i.e., EGF,^28^^,^^29^ can provide a new class of inhibitors with potential synergistic effects when combined with existing drugs. However, the cross-activity of more than one EGF-family ligand against the EGFR (and its related receptors, particularly the HER4 receptor) complicates this endeavor. For instance, the heparin-binding EGF-like growth factor (HB-EGF), transforming growth factor α (TGF-α), and amphiregulin (AR) also play roles in activating these receptors and promote cancer progression.^30^

The multiligand nature of EGFR signaling is thus better tackled through the development of polyspecific binders capable of quenching more than one growth factor, ideally, with high affinity. Previous attempts to engineer a recombinant form of the entire extracellular segment of the EGF and HER3 receptors could achieve broad ligand-binding specificity.^30^^,^^31^ These binders were constructed as dimeric IgG1 Fc-fragment fusions with the four extracellular domains of the receptor. While achieving broad inhibition of EGFR and HER3 ligands, these constructs require recombinant expression in mammalian cells and possess a large molecular weight of approximately 190 kDa, which can hamper their bioavailability at the relevant tumor tissue.^32^

**Figure 2:**
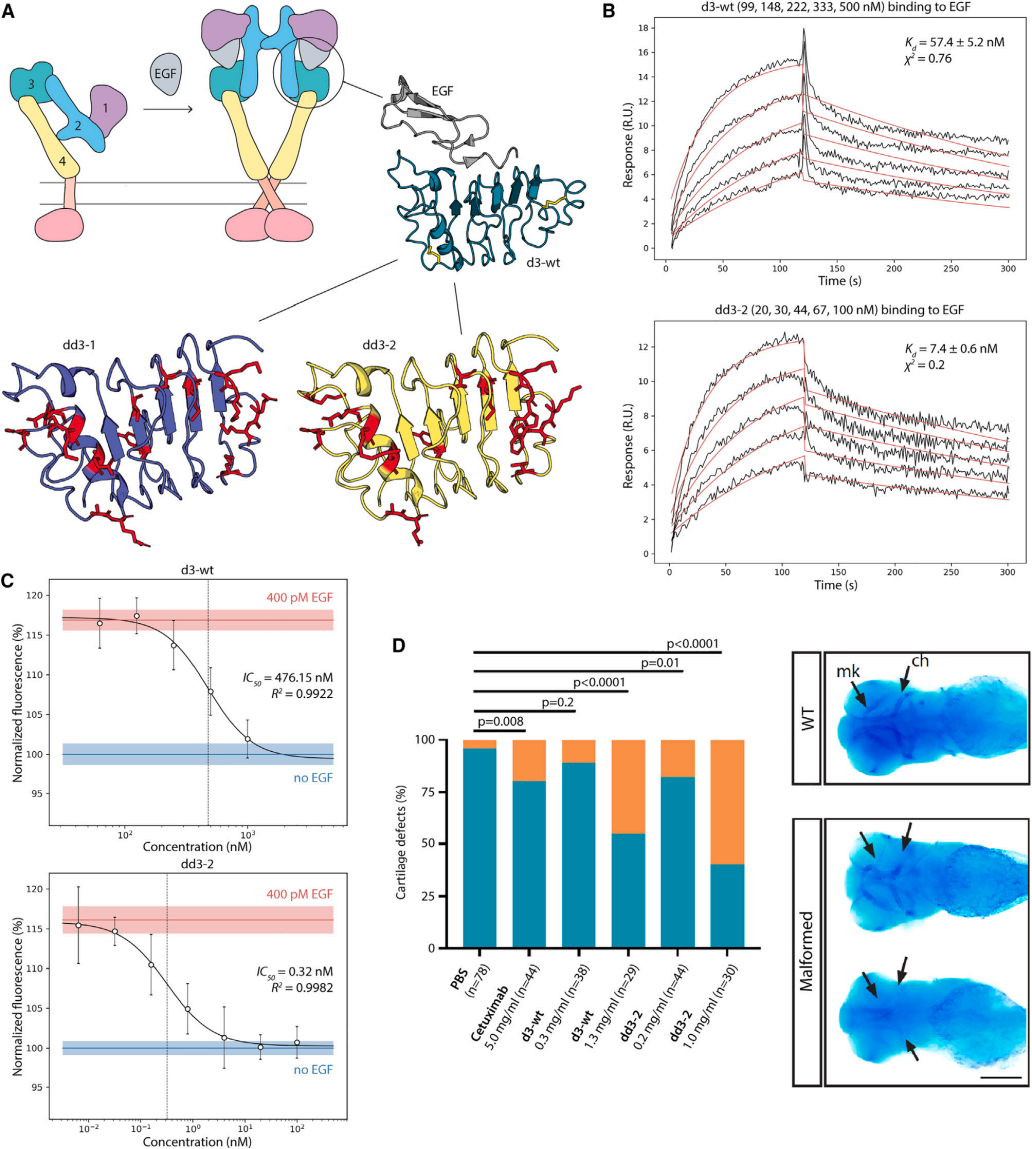
The design and characterization of EGFR inhibitors (A) The EGFR extracellular segment consists of four domains (d1, violet; d2, cyan; d3, teal; d4, yellow). In the absence of a ligand, it lies in a closed monomeric configuration. Upon ligand binding (here EGF, gray) the receptor adopts an open, dimeric configuration triggering intracellular signaling. As the third domain of the EGFR is reported to hold most of the binding affinity to the EGF ligand, it was used as a template to design soluble EGF binders. A close-up view of the EGF in complex with the wild-type d3 domain (d3-WT) is shown in gray and teal, respectively (PDB: 1IVO). Disulfide bridges in the d3-WT structure are shown as yellow sticks. Using the described energy function, the highest energy residues were identified. These residues were defined as mutable (shown in red) and designed using the combinatorial sampler. Two design models (dd3-1, purple; dd3-2, yellow) were finally chosen for experimental characterization. (B) SPR sensograms show the dd3-2 design to bind EGF tighter than d3-WT. A similar pattern with improved affinity of the design was observed toward other EGFR ligands (Figures S6 and S7). *K*_*d*_ is represented as the mean ± standard deviation (SD). The *χ*^2^ value represents the difference between the experimental data and the fitted curve averaged over the whole sensogram. Experimental data, black; fit, red. (C) Proliferation inhibition assays were done using the EGFR signaling-dependent A431 cells. The inhibition of cell proliferation was observed to be much stronger for dd3-2 (IC_50_ = 0.32 nM) than for d3-WT (IC_50_ = 476 nM). The positive and negative control values of cell proliferation with (400 pM) and without EGF treatment are indicated by red and blue lines, respectively. Shades and error bars represent SD across three replicates. (D) Pharyngeal skeleton of zebrafish embryos was stained with Alcian blue. First-arch Meckel’s cartilage (mk) and second-arch derivative ceratobranchials (ch) are observable (arrows). Upon EGFR inhibition, embryos with partial absence of Meckel’s cartilage and ceratobranchials or without any cartilage formation are observed and categorized in the malformed class. Cartilage defection upon injection of PBS, cetuximab, dd3-2, and d3-WT is shown in percentages for each group. Two-tailed p values were analyzed by a 2 × 2 contingency table in GraphPad. n, the number of evaluated embryos. Scale bar, 250 μm.

In this study, we aimed to create a miniature binder, using only one of the EGFR ligand-binding domains as a starting template and stabilizing it in its ligand-bound conformation by sequence redesign. Previous work has shown the human EGFR domain 3 (herein referred to as d3-WT) to be the ectodomain encoding most of the binding information to EGF.^33^ The d3-WT template sequence (Table S2) was restricted to a stretch of 168 amino acids, which contains disulfide bridges at the beginning and the end of the domain. The designs were instead based on a truncated domain boundary to include only 160 amino acids. All cysteine residues were excluded to improve downstream properties of the designs. The designable positions were set to comprise all residues with energy higher than a set threshold, which were identified using the “repack all” (ra) protocol. Running 100 instances of the tree swarm combinatorial sampler (cs_f2m2f) with randomized order of the designable positions yielded about 200 decoys with unique sequences. These were subject to accelerated MD (aMD) simulations and were ranked according to their conformational stability. The conformational stability scores as well as the RMSF plots derived from the latter simulations indicated a better stability of the designs compared with the d3-WT model, where the cysteines were reduced (Figure S5A). The two most mutually distant sequences in the top 10 designs were eventually selected for experimental evaluation, named dd3-1 and dd3-2 (designed domain 3; Figure 2A).

The starting template (d3-WT) and two designs (dd3-1 and dd3-2) were expressed in *E. coli.* Double purification from the soluble fraction showed the three proteins to have similar yields of approximately 0.2 mg per liter of culture. The designs also showed thermostability similar to that of d3-WT, as evaluated by nanoscale differential scanning fluorimetry (nanoDSF) (Figure S5B). However, the designs exhibited much stronger EGF inhibition activity in proliferation assays using the EGF-dependent epidermoid carcinoma cell line A431. Particularly, dd3-2 was the most active design, with a proliferation inhibition IC_50_ more than 1,000-fold lower compared with d3-WT (IC_50,dd3-2_ = 0.32 nM vs. IC_50,d3-WT_ = 476 nM) (Figure 2C) and only 3-fold higher than that of cetuximab, a therapeutic anti-EGFR antibody^34^ (Figure S5C). Next, to evaluate the difference in binding affinities toward EGF, we carried out surface plasmon resonance (SPR) titrations of our binders against immobilized EGF. The results showed that dd3-1 and dd3-2 bind EGF around 6-fold tighter than d3-WT, where dissociation constants (*K*_*d*_) were 10, 9, and 56 nM for dd3-1, dd3-2, and d3-WT, respectively (Table 1; Figures 2B, S5D, S6, and S7). This enhanced binding can be the result of stabilizing the ligand-bound conformation. Compared with previous results that weaponized an Fc chimera of the entire EGFR extracellular segment (∼95 kDa per subunit),^31^ our dd3-2 design is far smaller (18 kDa), leading to a better protein efficiency (i.e., ${\Delta G}_{bind}/MW$^35^) of the latter (−2.6 kJ/kDa) vs. the former (−0.5 kJ/kDa). To further evaluate the ability of our designs, particularly dd3-2, to bind other related EGFR ligands, we performed SPR binding experiments against HB-EGF and TGF-α, which are also important therapeutic targets for treatment of EGFR-dependent cancers.^30^ The results showed dd3-2 to bind HB-EGF 10-fold tighter than d3-WT, where *K*_*d*_ values of 35 and 370 nM were observed for dd3-2 and d3-WT, respectively, and demonstrated dd3-2 to bind TGF-α 11-fold tighter than d3-WT, with *K*_*d*_ values of 232 and 2,550 nM, respectively (Table 1; Figures S6 and S7). This polyspecificity of d3 proteins could be an explanation of their high inhibitory activity against the A431 cell line, given the complex signaling interplay among autocrine and paracrine EGFR ligands.^36^ In addition, the stronger inhibition by the designs can be attributed to their improved stability in the monomeric form in solution, as observed during the proteins’ purification and analytical size exclusion (Figure S8).

**Table 1:** SPR-derived binding parameters of d3-WT, dd3-1, and dd3-2 to different EGFR ligands

	$k_{a}$ (1/Ms)	$k_{d}$ (1/s)	$K_{D}$ (nM)
**d3-WT**			
EGF	$49.7\times 10^{3}\;\pm 0.7\times 10^{3}$	$2.8\times 10^{-3}\;\pm 0.2\times 10^{-3}$	56±2.1
HB-EGF	$47.2\times 10^{3}\;\pm 4.4\times 10^{3}$	$10.7\times 10^{-3}\;\pm 3.7\times 10^{-3}$	370.2±256.3
TGF-α	$38.4\times 10^{3}\;\pm 26.9\times 10^{3}$	$57.7\times 10^{-3}\;\pm 20.9\times 10^{-3}$	$2.55\times 10^{3}\;\pm 2.52\times 10^{3}$
**dd3-2**			
EGF	$248.7\times 10^{3}\;\pm 53.7\times 10^{3}$	$2.2\times 10^{-3}\;\pm 0.1\times 10^{-3}$	9.2±2.6
HB-EGF	$162.7\times 10^{3}\;\pm 68.5\times 10^{3}$	$4.3\times 10^{-3}\;\pm 0.8\times 10^{-3}$	34.5±8.1
TGF-α	$181.6\times 10^{3}\;\pm 22.6\times 10^{3}$	$37.8\times 10^{-3}\;\pm 12.2\times 10^{-3}$	231.9±54.6
**dd3-1**			
EGF	$260.2\times 10^{3}\;\pm 43.6\times 10^{3}$	$2.7\times 10^{-3}\;\pm 0.08\times 10^{-3}$	10.4±1.5

Sensograms are shown in Figures 2B, S5D, S6, and S7.

To investigate potential effects of the designed inhibitors *in vivo*, we injected equal volumes of PBS solution containing cetuximab (positive control), d3-WT, or dd3-2 into zebrafish embryos. As a negative control, pure PBS was injected. Inhibitors were administered starting at 4–6 h post-fertilization during 4 days. As a first step, the survival of the embryos was determined every day from 1 to 4 days post-fertilization (dpf). While almost no effect on survival was observed at any time point following injection of PBS, injections of cetuximab (5 mg/mL), d3-WT (0.3 mg/mL, 1.3 mg/mL), or dd3-2 (0.2 mg/mL, 1.0 mg/mL) were found to be lethal to different extents (Table S3). Next, we evaluated the morphological defects present in the surviving embryos at 4 dpf. Since it has been previously shown that EGFR inhibitors cause developmental defects in head cartilage,^37^ we analyzed head cartilage formation by Alcian blue staining (Figure 2D). In comparison to wild-type embryos with completely formed cartilaginous elements of the pharyngeal skeleton, the embryos with cartilaginous defects or even without any cartilage formation were classified as the malformed group. The results indicate cetuximab, d3-WT, and dd3-2 to affect skeletal development in a manner typical of EGFR signaling impairment. In line with the above-described biophysical and cell-based experiments, dd3-2 caused stronger effects in zebrafish embryos compared with d3-WT. Interestingly, both d3-WT and dd3-2 were more active than the anti-EGFR antibody cetuximab (Figure 2D).

### Design and characterization of copper binders

To further test the performance of the Damietta potential, we sought to design metallic radionuclide-binding proteins. Metal-binding proteins serve essential functions, including catalysis, sensing, transport, and storage.^38^ Designed metalloproteins can be tailored to encode one or more of such functions and be useful for a range of biomedical applications.^39^^,^^40^ Particularly, metalloproteins capable of high-affinity metal binding, efficient storage, and transport can serve as electron microscopy contrast agents,^41^ probes for magnetic resonance imaging,^42^ or targeted radioactive tracers for radiotherapy and diagnostic imaging purposes.^43^ We specifically aimed to design proteins to bind the radioactive ^64^Cu^2+^ ions. Such genetically encodable radiotracers can be fused with targeting proteins for high-resolution PET imaging or radioligand therapy with the therapeutic radioisotope ^67^Cu, forming an ideal therapeutic/diagnostic pair.^44^

**Figure 3:**
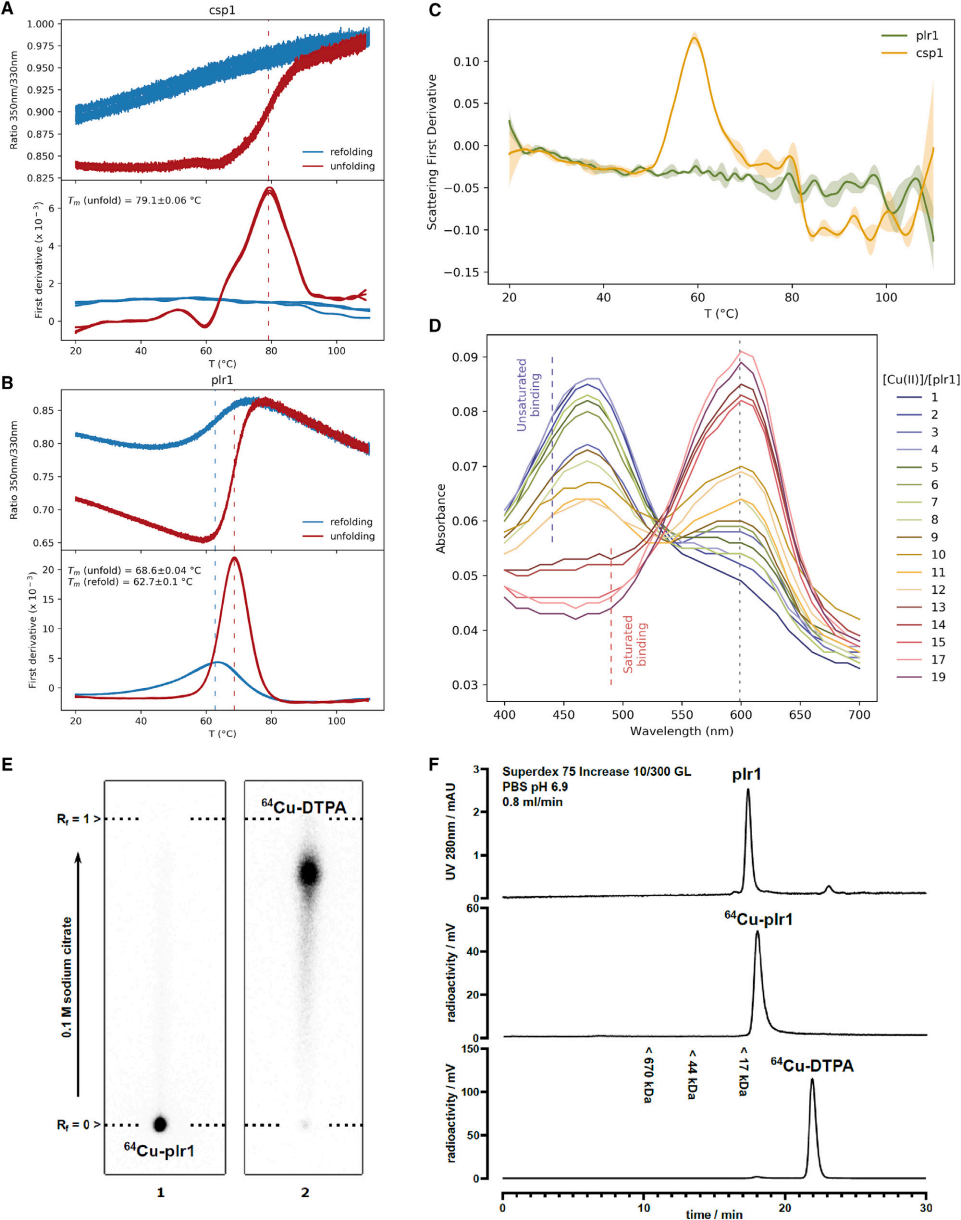
Design of stabilized ^64^Cu^2+^ binding proteins (A) NanoDSF melting curves show Csp1 to unfold at 79°C (red curves), without a refolding transition upon cooling (blue curves). (B) The plr1 design has a lower melting temperature of 69°C (red curves) but refolds upon cooling (blue curves). Melting temperatures (*T*_*m*_) are represented as the mean ± SD. (C) Csp1 scattering signal, however, shows an aggregation mid-point at 60°C, highlighting its colloidal instability, while plr1 does not show a change in scattering and does not precipitate in solution. Shading represents SD across three replicates. (D) Titrations performed using the chromophoric change of zincon indicate plr1 to bind between 12 and 13 Cu^2+^ ions per molecule. (E) Radiographic images of silica TLC plates with ^64^Cu^2+^-loaded plr1 (1) and the same sample stripped with DTPA for 3 h (2) show plr1 to bind ^64^Cu^2+^. TLC plates were developed with 0.1 M sodium citrate (pH 5). Proteins stay at the starting spot and DTPA migrates near the front line under these conditions. Similar results were observed for neg1 (Figure S9A). (F) Size-exclusion chromatogram of plr1 at 280 nm (top), radioactive signal of runs with ^64^Cu^2+^-loaded plr1 (middle), and a sample stripped with DTPA (bottom). plr1 binds ^64^Cu^2+^ and elutes corresponding to the same size as non-loaded plr1.

Given this intended function, we based our designs on helical bundles to create modules with robust and stable folding and thus minimal interference with any fused homing protein (e.g., tumor cell-targeting antibody fusions).^45^ We chose a cysteine-rich helical bundle protein (Csp1) as a starting template, which was shown to bind 13 Cu^+^ ions along its core.^46^ Although Csp1 has a low molecular weight (13 kDa) and possesses a simple up-down four-helix structure, it suffers several drawbacks. Specifically, Csp1 is unstable, is tetrameric, has low bacterial production yield, and has complex purification requirements.^46^ We therefore redesigned 22 amino acid positions, which were mostly surface exposed, to disrupt the oligomerization interface of the tetrameric Csp1 template and improve solubility and stability of the helical bundle (STAR Methods). The two most conformationally stable designs as assessed by MD simulations (named plr1 and plr2) were selected for experimental characterization (Table S2). The synthetic genes encoding plr1, plr2, and Csp1 were cloned without purification tags in a vector for expression in *E. coli*. The soluble expression levels were highest for plr1, followed by plr2, both being higher than Csp1. In contrast to the template, which has a net negative charge of −2, the designs possessed high net-positive charges (plr1, +14; plr2, +17). This particularly facilitated the purification of the supercharged designs using ion-exchange chromatography. We restricted our further characterization to the plr1 design given its high purification yield of >50 mg per liter of culture (>20-fold higher than Csp1). Analytical size exclusion showed plr1 to be monomeric, in contrast to the template Csp1, which was tetrameric and showed significant aggregation (Figure S8). Thermostability analysis indicated Csp1 and plr1 to have melting temperatures ($T_{m}$) of 79°C and 69°C, respectively (Figures 3A and 3B). However, Csp1 displayed a lower aggregation temperature ($T_{agg}$), which was 60°C and >110°C for Csp1 and plr1, respectively (Figure 3C). Similarly, irreversible thermal denaturation was observed for Csp1, in contrast to the reversible folding of plr1 (Figures 3A and 3B). The colloidal stability of the plr1 design is important for its clinical usefulness, given that aggregation tendency can greatly reduce the efficacy and raise the immunogenicity risk of biopharmaceuticals.^47^ The difficulty of handling Csp1 protein restricted our further functional analysis to the designed forms only.

By titrating copper and using a chromophoric probe, we observed a mid-point indicating approximately 13 Cu^2+^ binding sites on plr1 (Figure 3D). This high metal-binding capacity of almost 1 metal/kDa of protein is consistent with that originally reported by Csp1.^46^ This binding ratio could be beneficial for high imaging sensitivity and efficacy in radiotracer imaging and radioimmunotherapy applications, respectively. To test suitable labeling conditions with ^64^Cu^2+^, we incubated plr1 with the buffered radioisotope at a specific radioactivity of 2 GBq/mg. After incubation for 60 min at 35°C, the radioactivity was efficiently (>90%) sequestered into plr1, as judged by radio-thin-layer chromatography (radio-TLC) (Figure 3E), and eluted from size-exclusion chromatography with the same profile as unlabeled plr1 (Figure 3F). These results strongly support the capacity of the design to readily and stably chelate copper radioisotopes through a simple incubation procedure.

**Figure 4:**
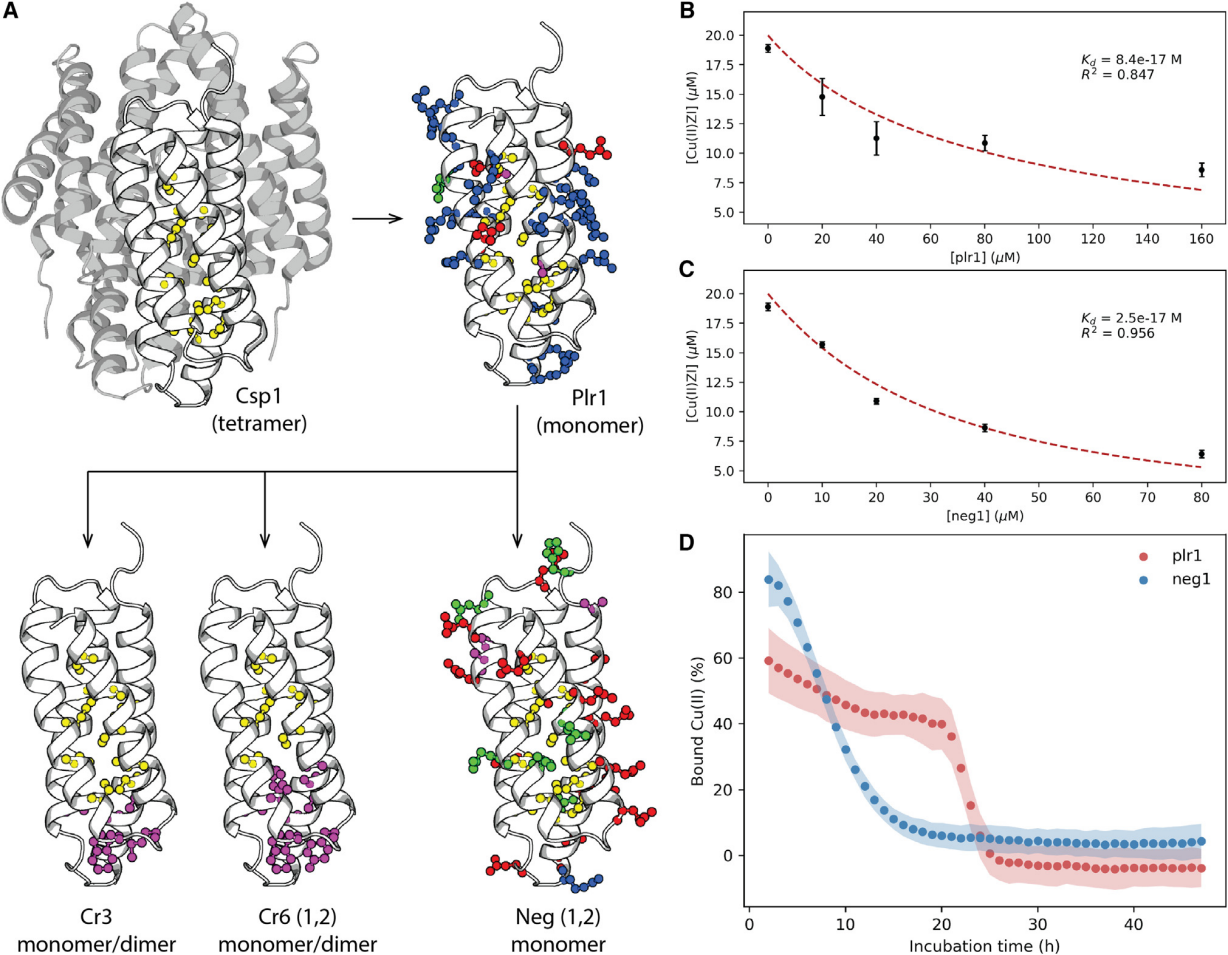
Exploring the sequence-stability relationship of copper-binding proteins (A) A phylogeny of the redesigned copper-binding proteins. The Csp1 template is shown in white in its tetrameric configuration (gray protomers) and the cysteine side chains at the core are depicted in a ball-and-stick representation (PDB: 5FJE). The polar side chains introduced in the first-generation design (plr1 model) are shown, yielding a monomeric, positively supercharged protein. Starting from the plr1 sequence, second-generation designs belong to three classes: core-repacked designs where either three (cr3) or six (cr61 and cr62) core cysteine residues were eliminated or negatively supercharged designs (neg1, neg2). Side chains colored yellow are cysteines, blue are positively charged, red are negatively charged, green are polar, and purple are non-polar. (B and C) Competitive Cu^2+^ binding assays using zincon for plr1 and neg1 designs show sub-femtomolar dissociation constants, whereby neg1 binds Cu^2+^ more than 3-fold tighter than plr1. Error bars represent SD across two replicates. (D) The Cu^2+^ release upon incubating protein:Cu^2+^ complexes in 4-fold diluted serum. Plr1 remains associated with Cu^2+^ despite the lower initial affinity, followed by cooperative dissociation. neg1, on the other hand, displays higher initial affinity, but faster Cu^2+^ dissociation. This highlights the higher proteolytic resistance of plr1 compared with neg1, which corresponds to their expected thermostabilities (Figures 3B and S9B). Shading represents SD across three replicates.

Exploring the determinants of structural stability of the copper-binding proteins can guide the generation of enhanced variants for clinical applications. Through a second design round, we aimed to create two classes of variants to evaluate their metal-binding stability. The first class involved repacking core residues to eliminate three or six core cysteine residues, cr3, and cr61 or cr62 designs, respectively (Figure 4A; Table S2). These variants have their cysteine-lined lumen plugged at the solvent-accessible end, which could restrict the outward diffusion of coordinated metal ions. The second class was negatively supercharged designs (neg1 and neg2), where the positively charged residues of plr1 were forcibly redesigned into neutral or negatively charged residues (Figure 4A; Table S2). These variants would provide a favorable electrostatic environment for the coordinated metal ions, especially given the +2 oxidation state of the target copper ions. While the five new designs were all well expressed, the three repacked core variants (cr3, cr61, and cr62) were majorly dimeric in solution and therefore were excluded from further analysis. Conversely, the neg1 variant could be purified in a monomeric state. In radio-TLC experiments, neg1 bound ^64^Cu^2+^, which was also observed for plr1 (Figure S9A). Competitive binding assays showed neg1 to bind Cu^2+^ 3-fold tighter compared with the plr1 design (Figures 4B and 4C), pointing to the possible stabilization of the metal:protein complex by negative charges. However, the thermal stability of the neg1 apoprotein decreased in comparison to plr1, where an earlier melting transition was observed for neg1, despite its reversible unfolding (Figure S9B). This affinity/stability trade-off was also evident when the copper-binding stability was assessed in untreated fetal bovine serum *in vitro* (Figure 4D). Whereas neg1 initially bound more copper ions than plr1, it released copper faster. Fitting to a first-order decay model yields dissociation rate constants in 4-fold diluted serum of $0.094\;h^{-1}$ and $0.054\;h^{-1}$ for neg1 and plr1, respectively. Notably, plr1 displayed a more complex copper dissociation behavior with a possible cooperative dissociation step, which might be due to accelerated chemical degradation upon copper ion release. These results guide further, more detailed, investigation of protein charge tuning and *trans*-chelation to maximize the binding stability of the radioisotope.

Radiolabeling of proteins (e.g., tumor-targeting antibodies) for PET imaging is not only important for routine tumor-imaging applications, but also for tracking the biodistribution of protein- and cell-based therapies *in vivo*. Currently, associating a metallic radionuclide (such as ^64^Cu^2+^) to a protein is mostly performed through chemical coupling of chelating agents (e.g., DOTA) to the protein of interest (e.g., NHS coupling).^48^ This undirected chemical coupling requires additional processing steps and introduces positional and stoichiometric heterogeneity of the labeled proteins, lowering their fidelity and usefulness for clinical applications. Conversely, our designed copper binders can be used as genetically encodable PET labeling tags that can be expressed on a target cell surface or as a single-chain fusion with the protein of interest. Given the high affinity of these proteins to Cu^2+^ ions, they can be readily loaded with copper radionuclides under mild conditions, greatly simplifying the radiolabeling procedure.

### Limitations of the study

Currently, the described framework is restricted to fixed-backbone sequence design. However, we foresee that further developments of the framework will allow it to support motion. Also, while in its current state the framework applies isotropic charges, implementing more advanced electrostatic potential to describe the rotamer library can greatly enhance the scoring accuracy, at no added run-time cost.

## STAR★Methods

### Key resources table

**Table:** 

REAGENT or RESOURCE	SOURCE	IDENTIFIER
**Antibodies**		

Cetuximab	MedChemExpress	Cat#HY-P9905

**Bacterial and virus strains**		

BL21(DE3) Competent Cells - Novagen	Sigma-Aldrich	Cat#69450

**Chemicals, peptides, and recombinant proteins**		

cOmplete, EDTA-free Protease Inhibitor Cocktail	Roche	Cat#5056489001
DNase I	PanReac AppliChem	Cat#A3778
DMEM, high glucose, pyruvate	Gibco	Cat#41966029
Fetal Bovine Serum, certified, heat inactivated	Gibco	Cat#10082147
DPBS, no calcium, no magnesium	Gibco	Cat#14190144
Alcian Blue 8 GX	Sigma-Aldrich	Cat#A5268
Zincon monosodium salt	Supelco	Cat#96440
Recombinant Human EGF	PeproTech	Cat#AF-100-15
Recombinant Human HB-EGF	R&D Systems	Cat#259-HE-050/CF
Recombinant Human TGF-alpha	R&D Systems	Cat#239-A-100

**Critical commercial assays**		

CellTiter-Blue® Cell Viability Assay	Promega	Cat#G8080

**Deposited data**		

Molecular dynamics simulation data for amino acids in explicit water performed under the CHARMM27 force field	Vitalini et al.^12^	ftp://bdg.chemie.fu-berlin.de/Ac-X-NHMe/
Thermodynamic stability data for protein G mutants	Nisthal et al.^18^	ProtaBank ID: gwoS2haU3
Protein folding stability data for protein mutants measured by cDNA display proteolysis	Tsuboyama et al.^19^	https://doi.org/10.5281/zenodo.7844779

**Experimental models: Cell lines**		

A431 Cell Line human	ECACC	Cat#85090402; RRID:CVCL_0037

**Experimental models: Organisms/strains**		

Zebrafish: wild-type: AB	N/A	RRID:ZIRC_ZL1

**Software and algorithms**		

Damietta	This paper	https://doi.org/10.5281/zenodo.8152656
NAMD	Phillips et al.^49^	RRID:SCR_014894
VMD	Humphrey et al.^50^	RRID:SCR_001820
Prism	GraphPad Software, Inc.	RRID:SCR_002798
PyMOL	Schrödinger, Inc.	RRID:SCR_000305
Biacore X100 Evaluation Software	Cytiva	RRID:SCR_015936

**Other**		

Gene synthesis	Synbio Technologies, Inc., BioCat GmbH	N/A
Millex-HV Filter, 0.45 μm, PVDF, 33 mm	Millipore	Cat# SLHV033RS
Amicon Ultra-15 centrifugal filter unit, 10 kDa	Millipore	Cat# UFC901024
HisTrap Excel column, 5 ml	Cytiva	Cat#GE17-3712-06
Superdex 75 Increase 10/300 GL column	Cytiva	Cat#29148721
HiTrap Capto Q column, 5 ml	Cytiva	Cat#11001303
HiTrap Capto S column, 5 ml	Cytiva	Cat#17544123
Prometheus Standard Capillaries	Nanotemper	Cat#PR-C002
Sensor Chip CM5	Cytiva	Cat#BR100012
96 Well TC-Treated Microplates	Corning	Cat#3596
UV-Star plate, 384 well, F-bottom	Greiner	Cat#781801

### Resource availability

#### Lead contact

Further information and requests for resources and reagents should be directed to and will be fulfilled by the lead contact, Mohammad ElGamacy (mohammad.elgamacy@med.uni-tuebingen.de).

#### Materials availability

This study did not generate new unique reagents.

### Experimental model details

#### Bacterial protein expression system

BL21(DE3) competent *E. coli* cells were used for transformation and high-level protein expression using a T7 RNA polymerase-IPTG induction system.

#### Cell line

A431 cells (ECACC 85090402; RRID: CVCL_0037) were cultured in DMEM medium supplemented with 10% fetal bovine serum (FBS), at 37 °C, 5% CO_2_. Sub-confluent cultures were split 1:10 twice a week.

#### Zebrafish

Wild-type zebrafish line (AB, RRID:ZIRC_ZL1) was used for the experiments. Treatment of zebrafish embryos with tested inhibitors started at 4-6 hpf and continued until 4 dpf. The sex of the embryos was not taken into consideration. Zebrafish were maintained according to standard protocols and handled in accordance with European Union animal protection directive 2010/63/EU and approved by the local government (Tierschutzgesetz §11, Abs. 1, Nr. 1, husbandry permit 35/9185.46/Uni TÜ).

### Method details

#### The rotamer library

Large-scale molecular dynamics simulations of capped amino acids (i.e. Ac-X-NHMe, where X represents the amino acid symbol) were used as conformational pools from which representative rotamers were sampled. These trajectories can be performed under a predefined force field in explicit water, yielding a pool of freely inter-changing conformations. In the current implementation, we have used a set of trajectories performed under the CHARMM27 force field in explicit solvent by Vitalini et al.^12^ The trajectory of every Ac-X-NHMe amino acid is 1-μs-long and was uniformly partitioned into 36 (φ,ψ) bins (within (±60, ±60) intervals). The propensity of each backbone conformational state (i.e. a (φ,ψ) bin) was used to represent its relative energy with respect to all of the other conformational bins as:

(Equation 1) $${\Delta G}_{pp}=-k_{b}T\;\ln\frac{O_{{\left(\varphi ,\psi \right)}_{m}}}{N}$$

Where $k_{b}$ is the Boltzmann constant in kcal·mol^−1^K^−1^, T is the temperature in Kelvins (here given as constant values of 0.001985875 and 298, respectively), $O_{{\left(\varphi ,\psi \right)}_{m}}$ is the number of observations in the m ^th^ (φ,ψ) conformation bin, and N is the total number of conformations. A similar explicit internal energy term is used to describe the side chain conformational preference, where all conformations within each ${\left(\varphi ,\psi \right)}_{m}$ bin are aligned to a single frame-of-references using the three atoms C^β^ (or Gly H^α1^), C^α^, N. In this frame of reference C^α^ is positioned at the origin, C^α^→C^β^ (or C^α^→H^α1^) vector is aligned along the z-axis, and the C^α^→N vector lies along the xy-plain. The aligned conformers undergo a 3D *k*-means clustering including all of their atomic coordinates, resulting in k representative side chain conformers at the center of each cluster. Here the values of k was set to 50, and 100 conformational clusters to build the 50- and 100-rotamer libraries, respectively. The representative conformer of each cluster was taken to be the one with the lowest RMSD to the average structure of the entire cluster, where the energy of every cluster is defined as:

(Equation 2) $${\Delta G}_{k}=-k_{b}T\;\ln\frac{O_{\left(k_{n}|{\left(\varphi ,\psi \right)}_{m}\right)}}{O_{{\left(\varphi ,\psi \right)}_{m}}}$$

Where $O_{\left(k_{n}|{\left(\varphi ,\psi \right)}_{m}\right)}$ is the number of observations in conformation bin ${\left(\varphi ,\psi \right)}_{m}$ of the n ^th^ cluster out of k clusters. In cases where the entire molecular dynamics simulation results in a conformational bin that is underpopulated, i.e. $O_{{\left(\varphi ,\psi \right)}_{m}}<k$, the entire bin is not represented in the library.

#### The energy function

The energy function is composed of 5 terms representing the energy difference to a ground state of a solvated, capped amino acid, as follows: backbone internal energy (${\Delta G}_{pp}$, Equation 1), side chain conformational energy (${\Delta G}_{k}$, Equation 2), Lennard-Jones interaction energy (${\Delta G}_{LJ}$), solvation energy (${\Delta G}_{solv}$), and electrostatic interaction energy (${\Delta G}_{elec}$). The total energy is a weighted sum of these terms, as:

(Equation 3) $${\Delta G}_{total}={w_{pp}\Delta G}_{pp}+{w_{k}\Delta G}_{k}+w_{LJ}\left(\Delta G_{LJ}-\Delta G_{LJ,ref}\right)+w_{solv}\left(\Delta G_{solv}-\Delta G_{solv,ref}\right)+w_{solv}\left(\Delta G_{elec}-\Delta G_{elec,ref}\right)$$

The energy calculation scheme follows a two-body formulation whereby the interactions are only calculated between two sets of atoms belonging to the *1*^*st*^*-body* and the *2*^*nd*^*-body*. The *1*^*st*^*-body* atoms are all the atoms within a bounding box of dimensions $d_{box}\times d_{box}\times d_{box}$ excluding the side chain atoms of the mutable residue, where the bounding box is centered at the Cα atom of the mutable residue. The *2*^*nd*^*-body* atoms represent the side chains of the rotamer to be placed in the mutable residue position, as sampled from the rotamer library. This scheme applies to the interaction energy terms (${\Delta G}_{LJ}$ and ${\Delta G}_{elec}$) as well as the solvation free energy term (${\Delta G}_{solv}$). The non-bonded energy terms between the inbound sidechain atoms and the environment are corrected by subtracting a reference energy ($\Delta G_{LJ,ref}$, $\Delta G_{solv,ref}$, and $\Delta G_{elec,ref}$; Equation 3). These reference values describe the interaction energies between the side chain of a rotamer and its backbone atoms in the respective conformation pooled from the MD. These reference energy values are precomputed with the rotamer library and are subtracted from the final interaction energy before it is weighted (Equation 3). To preserve the compatibility between different energy terms, the partial charges and Lennard-Jones parameters are also obtained from the CHARMM27 force field parameters,^14^ and the surface-area based solvation energy term relied on the CHARMM-based parameters for the EEF1-SB model.^51^ Moreover, the ${\Delta G}_{pp}$ (Equation 1) and ${\Delta G}_{k}$ (Equation 2) terms, are derived from conformational distributions extracted from simulations that used the CHARMM27 force field.^12^

For evaluating electrostatic interaction energies, an approximation of the Generalised Born model was used. The interactions are calculated between the *1*^*st*^*-body* atoms i∈I of the protein where the mutable residue side chain atoms are deleted, and I protein atoms exist within a bounding simulation box, and the *2*^*nd*^*-body* atoms j∈J that constitute the inbound side chain atoms looked up from the rotamer library. The electrostatics function was composed of three terms as follows:

(Equation 4) $${\Delta G}_{elec}=\frac{332q_{i}q_{j}}{\varepsilon \left(r\right)r_{ij}}\left(1-\frac{\varepsilon_{p}\bar{r}}{{\left({\bar{r}}_{ij}^{2}+{\bar{b}}^{2}e^{-\frac{{\bar{r}}_{ij}^{2}}{4{\bar{b}}^{2}}}\right)}^{\frac{1}{2}}}\left(\frac{1}{\varepsilon_{p}}-\frac{1}{\varepsilon_{s}}\right)\right)-166\frac{q_{i}^{2}}{\bar{b}}\left(\frac{1}{\varepsilon_{p}}-\frac{1}{\varepsilon_{s}}\right)$$

Where $q_{i}$ and $q_{j}$ represent the partial charges of atoms i and j, separated by the distance $r_{ij}$. A distance-dependent dielectric function of ε(r)=r was assumed. As the electrostatic interactions cutoff was set to 7.0 Å, the value of ε(r) ranged as $\sigma_{LJ}<\varepsilon \left(r\right)<7.0$, where $\sigma_{LJ}$ of a carbon-carbon interaction, for example, is approximately 4 Å. $\varepsilon_{p}$ and $\varepsilon_{s}$ represent the dielectric constant of protein core ($\varepsilon_{p}=8)$ and water ($\varepsilon_{s}=75$), respectively.

Equation 4 was derived in this form to reduce the computing cost, whereby the first term is precomputed for 2^nd^ body as $\frac{322q_{j}}{\varepsilon \left(r\right)r}$, that is multiplied by the 1^st^ body partial charges tensor (i.e. $q_{i}$), which is computed rapidly on the fly. The second and third terms represent the charge solvation corrections according to the Generalised Born model and are computed as a tensor scalar and an additive term, respectively. In the second term, the average interatomic distance in the simulation cube ${\bar{r}}_{ij}^{2}$ was used instead of the individual distances, and born radius was taken as the average born radius in the simulation cube; $b_{i}=b_{j}=\bar{b}$. The average interatomic distance within the simulation cube ${\bar{r}}_{ij}$ was set to ${0.6617d}_{box}$, while the average born radius $\bar{b}$ was approximated according to the fraction of filled volume of the simulation cube (i.e. $d_{box}\frac{V_{filled}}{V_{total}}$).

The Lennard-Jones function was implemented as a piece-wise function to avoid sensitivity to interatomic clashes, which can be relaxed upon MD-based minimization. The piece-wise function consists of three components; a standard LJ term in the attractive range of inter-atomic distances, a slow-growing repulsive term across a band of the atomic crust, and a flat maximum at a defined atomic core, as follows:

(Equation 5) $${\Delta G}_{LJ}=\left\{ \begin{array}{c} \varepsilon_{LJ,j}\left(\frac{\sigma_{LJ,j}^{12}}{r_{ij}^{12}}-\frac{\sigma_{LJ,j}^{6}}{r_{ij}^{6}}\right)\;\left(\sigma_{LJ,j}-c_{soft}\right)<r_{ij}\leq c_{lr}\\ \varepsilon_{LJ,j}\frac{\sigma_{LJ,j}^{2}}{r_{ij}^{2}}\;\left(\sigma_{LJ,j}-c_{hard}\right)<r_{ij}\leq \left(\sigma_{LJ,j}-c_{soft}\right)\\ \varepsilon_{LJ,j}\frac{\sigma_{LJ,j}^{4}}{{\left(\sigma_{LJ,j}-c_{hard}\right)}^{4}}\;0<r_{ij}\leq \left(\sigma_{LJ,j}-c_{hard}\right) \end{array}\right.$$

Where $\varepsilon_{LJ,j}$ and $\sigma_{LJ,j}$ are the minimum LJ energy value (in kcal·mol^−1^) and the LJ radius (in Å) of atom j as obtained from the CHARMM27 parameters.^14^ The cutoffs $c_{soft}$ and $c_{hard}$ were set to 0.25 Å and σLJ,j2, respectively. The use of $\varepsilon_{LJ,j}$ and $\sigma_{LJ,j}$ parameters of atom j instead of the averaged parameters for atoms i and j was aimed at lowering the computing cost, since the entire LJ interaction fields are pre-calculated for inbound rotamer atoms (i.e. j∈J atoms). Both the LJ and electrostatic interaction fields are calculated for all values of $0<r_{ij}\leq c_{lr}$ at a resolution of 0.5 Å, where $c_{lr}$ is the long-range cutoff that is set to 7.0 Å. Such fields are stored in the chemical library provided with the software, and are looked up during the design process depending on the mutable position (φ,ψ) bin.

These interactions are calculated within a cube where a single side of the cube ($d_{box}$) has the size of 22Å, containing 44×44×44 voxels, where the voxel resolution is 0.5Å. To evaluate the impact of varying the voxel resolution on the calculation accuracy, we emulated the interaction of carbon atoms at varying resolutions. The results showed most of the energy error to stem from the repulsive part of the function (i.e. $r_{ij}<\sigma_{LJ,ij}$), which was largely positive in value as $r_{ij}$ is floored to the nearest discrete bin (Figure S10A). The results also showed the energy error to substantially decrease above a resolution of 0.4Å (Figure S10A). It worth highlighting that this approach is dissimilar to other parallel energy computations that are primarily thread-based, where a constant set of instructions can access a large number of shared variables (here; atom attributes) across the main (or a GPU) memory.^52^ Instead, the presented framework simplifies these calculations by encoding most of the energy function information into smooth fields associated with each discrete rotamer (Figure S10B). This leaves only populating the positions of the environment atoms as the quick on-the-fly step that is performed once for each designable position (Figure S10C). This renders the total energy much faster to compute through only 2 instructions and 2 variables; as $E_{LJ}=\sum \left(v_{i,full}⊙v_{j\in J}\right)$ (Figure S10D). Our method further stacks all rotamer fields belonging to one side chain into a single variable (i.e. 4D tensors encoding $100\;rotamers\times \dim_{x}\times \dim_{y}\times \dim_{z}$) in order to reduce the number of memory calls. Unlike the standard way of computing the LJ function, this operation avoids any exponentiation or square roots, and offers substantially higher arithmetic intensity.

A generic solvation free energy term based on a surface area method was also put in place to account for the hydrophobic effect. This term was adapted from the EEF1-SB energy model parameters,^51^ and follows the form:

(Equation 6) $${\Delta G}_{solv}=\sum_{l}\sigma_{solv,l}A_{l}\left(r_{l}\right)$$

Where $\sigma_{solv,l}$ is the solvation energy per unit surface area (in kcal·mol^−1^Å^−2^) of atom l, with solvent-exposed surface area $A_{l}$ when located at position vector $r_{l}$. An approximation of the $A_{l}\left(r_{l}\right)$ function is derived from the non-occluded vdW surface area of slightly inflated vdW radii. This radial inflation was performed here by an added 0.5 Å to the atomic vdW radii, while correcting for the $\sigma_{solv,l}$ for the larger atomic surface area to keep the solvation energy per atom type constant. The implementation relied on a voxelized representation of atomic crusts encoding the $\sigma_{solv,l}$ values in tensorial forms as well as core-masking tensors which are used to exclude the occluded atomic surfaces. This energy term is calculated separately for the *1*^*st*^*-body* atoms (${\Delta G}_{solv,1}$), the *2*^*nd*^*-body* (${\Delta G}_{solv,2}$), and the *1*^*st*^*-body* and *2*^*nd*^*-body* combined (${\Delta G}_{solv,1,2}$). These values would represent the solvated free energies of the protein environment with the removed side chain atoms at the mutable residue, the inbound side chain atoms of a rotamer from the rotamer library, and the combined protein environment and rotamer side chain atoms after rotamer placement, respectively. Given these three values, the final solvation free energy term is calculated as follows:

(Equation 7) $${\Delta G}_{solv}={\Delta G}_{solv,1,2}-\left({\Delta G}_{solv,1}+{\Delta G}_{solv,2}\right)$$

Finally, while the different terms of the energy function are compatible as they were derived from the same force field, the softening of the repulsive component of the LJ term necessitates the downscaling of the electrostatic term ($w_{elec}$ = 0.25), in order to avoid highly clashing configurations with optimal electrostatic interactions. Additionally, the $w_{k}$ is recommended to be set to 0 for mutagenesis tasks, and to 1 for repacking tasks. This is as ${\Delta G}_{k}$ is not directly comparable across different amino acid types, given that different amino acid types have drastically different chemical exchange timeframes in solution, even when their backbone is fixed.^53^ Otherwise, the other weighting factors were all set to 1.0; $w_{pp}$ = $w_{k}$ = $w_{LJ}$ = $w_{solv}$ = 1.0. Additionally, to deploy some tiered scoring to avoid calculating all energy terms for highly clashing rotamers, a maximum LJ energy value was set to 5.0 kcal·mol^-1^.

#### Evaluation of scoring accuracy against stability benchmarks

Ability of the framework to evaluate stability of protein mutants was benchmarked using five independent previously reported experimental dataset of: 1) mutants of the β1 domain of streptococcal protein G (PDB: 1PGA);^18^ 2) mutants of the N-terminal domain of phage 434 repressor (PDB: 1R69); 3) mutants of the SH3 domain in human obscurin (PDB: 1V1C), 4) mutants of the N-terminal domain of ribosomal protein L9 (PDB: 2HBB), and 5) mutants of the r11_829_TrROS protein designed by trRosetta hallucination (an AlphaFold model of the design was used).^19^ Generating mutants and estimating their energies was done using a single-point (sp) routine with the parameters (-max_lj 10.0, -w_pp 1.0, -w_k 1.0, -w_lj 1.0, -w_solv 1.0, -w_elec 0.25). ΔΔ*G* was calculated as the difference in free energy between the mutant and the wild type reference. Predictive potential of a software was assessed using a Pearson correlation coefficient (R) for computed energy values against experimental data. For the dataset of Gβ1 mutants, performance of Damietta was analyzed in comparison with performance of Rosetta framework (ddg_monomer application, NoMin protocol) described before by Nisthal et al.^18^

#### Evaluation of rotamer and sequence recovery

For testing the ability of Damietta single-point mutagenesis routine to recover native side-chain conformations (rotamer recovery) the dataset described by Zhou et al.^6^ was used. The dataset consists of 9 pairs of structures, where each pair represents one X-ray and one NMR structure of the same monomeric protein (PDB: 1TVG, 1XPW; 3C4S, 2JZ2; 2O0Q, 2JQN; 2Q00, 2JPU; 3IDU, 2KL6; 3K63, 2KRT; 3FIF, 2JN0; 3H9X, 2KFP; 1TTZ, 1XPV). For NMR entries, first model was used (out of twenty models in each ensemble). For each structure in the dataset, the repack all (ra) routine was run for 10 successive rounds, where side chains of all amino acids were repacked from N- to C-terminus in each round. Rotamer recovery was evaluated in terms of predicted χ_1_ and χ_2_ side-chain torsion angles. An angle prediction was considered correct if the torsion error was in the range of ±40° from the native angle.^54^ χ_1_ accuracy was defined as a percentage of residues within a protein with correctly predicted χ_1_ angle. χ_1&2_ accuracy was defined as a percentage of residues within a protein with correctly predicted both χ_1_ and χ_2_ angles. Results were presented as a boxplot, where lower and upper hinges represent first and third quartile, respectively, and the whiskers extend from the box by 1.5 times the inter-quartile range (Figure S3). Core residues were identified as residues with solvent exposed surface area less than 5 Å^2^.

To evaluate the recovery of native amino acid identities (sequence recovery) a dataset of 9 above-mentioned X-ray structures was used (PDB: 1TVG, 3C4S, 2O0Q, 2Q00, 3IDU, 3K63, 3FIF, 3H9X, 1TTZ). Each amino acid position (except of N-terminal and C-terminal residues) was mutated individually using the single-point (sp) routine with the following parameters: 20 target amino acids, -max_lj 10.0, -w_pp 1.0, -w_k 1.0, -w_lj 1.0, -w_solv 1.0, -w_elec 0.25). Sequence recovery was calculated as a percentage of amino acid positions within a protein at which the lowest-energy residue selected by sp sampler is identical to the native amino acid.

#### Design of EGFR inhibitors

The design was performed using the EGF:d3 structure (PDB: 1IVO) as a template (residue range: 313-480 for d3-wt, and 313-472 for designed proteins). The input structure for Damietta applications has to be a single-chain structure that is CHARMM-typed, with all hydrogens included and no missing atoms. The input coordinates of the d3-wt domain structure was CHARMM-typed using the automatic PSF generation plugin (autopsf, version 1.8) as implemented in VMD (version 1.9.3).^50^ Using the repack-all application (damietta_ra), we had identified residues with energy higher than 20 kcal/mol, as well as all cysteine residues. These residues were subject to combinatorial design using the few-to-many-to-few sampler (damietta_cs_f2m2f). The combinatorial sampler mutates and moves the designable residues, and moves the repackable residues as specified in the spec file (Methods S1). The average energy per residue is calculated for both the mutable and repackable residues and are deterministically minimized. The order by which residues are optimized is randomizable and multiple traversals of the mutagenesis decision tree can be conducted. The context of the spec file contained the following parameters: the mutable residues (mut_res) and their target mutations; the repackable residues (rpk_res); a scrambled order of the mutable residues in every instance (scramble_order); the top mutations considered for combinatorial optimization (m_mutations); the number of parallel paths traversed down the decision tree (n_paths); the number of repeat iterations by which the tree is traversed (n_iters). The target mutations in the spec file were specified according to a sequence profile of d3 homologues obtained from closest 500 homologous sequences in the nr protein database. The spec file was run 100 instances, for 100 CPU hours/instance. The resulting decoys were further filtered according to their stability in accelerated molecular dynamics (aMD) simulations that follow a serial tempering routine previously described.^55^^,^^56^ These simulations were conducted using NAMD^49^ with a generalized Born implicit solvent model and a timestep of 1 fs. The tempering scheme starts by 500 steps of conjugate gradient minimization followed by an annealing cycle that was repeated for 160 rounds, with one configuration dump at the end of each cycle. The annealing cycle follows the sequence of: 100 minimization steps, 3000 timesteps (i.e. 3 ps) in a 370 K Langevin bath, 4000 timesteps (i.e. 4 ps) in a 250 K Langevin bath, and 100 minimization steps. The two most conformationally homogeneous designs (dd3-1 and dd3-2) were accordingly selected for experimental characterization. Conformational homogeneity was quantified as the average all-*vs.*-all RMSD averaged across all frames (i.e. 160 frames) output from the aMD simulations using VMD.^50^

#### Purification of EGFR inhibitors

Sequences of d3-wt and the designed proteins (dd3-1, dd3-2, Table S2) were ordered as synthetic genes in pET-28a(+) expression vector (Synbio Technologies, Inc.). Plasmids were transformed into chemically competent *E. coli* BL21(DE3) using the heat shock method. Transformed cells were grown in LB medium supplemented with 40 μg/ml kanamycin at 37 °C. At OD600 of around 0.6-0.8, cells were induced with 1mM IPTG and incubated overnight at 25°C for protein expression. Cells were harvested by centrifugation at 5000 g at 4°C for 15 min and lysed in 30 ml of lysis buffer (1M guanidinium chloride, 100 mM NaCl, 50mM Tris-HCl pH 8.0) supplemented with a tablet of the cOmplete, EDTA-free Protease Inhibitor Cocktail (Roche) and 3 mg of lyophilized DNase I (PanReac AppliChem) using a Branson Sonifier 250 (Fisher Scientific). The lysate was cleared by centrifugation at 28000 g at 4°C for 50 min and the supernatant was filtered through a 0.45 μm filter (Millipore). The sample was applied to a 5 ml HisTrap Excel column (Cytiva). The running buffer was 200 mM NaCl, 30 mM Tris-HCl pH 8.0. After sequential washing the column with 20 ml of the running buffer and 20 ml of the running buffer supplemented with 50 mM imidazole, fractions were collected by linear gradient elution using 150 mM NaCl, 30 mM Tris-HCl pH 8.0, 500 mM imidazole buffer. The eluted fractions containing the protein of interest were pooled, concentrated using 10 kDa MWCO centrifugal filters (Millipore), and further purified on a Superdex 75 Increase 10/300 GL gel filtration column (Cytiva) using PBS. Gel filtration fractions containing pure protein in the desired oligomeric state were pooled, concentrated, and stored at -20 °C for subsequent analyses. Both IMAC and gel filtration steps were performed on an Äkta Pure chromatography system (Cytiva).

#### Thermostability analysis of EGFR inhibitors

NanoDSF measurements using Prometheus NT.48 (Nanotemper) were performed to evaluate thermostability of d3-wt and the designs (dd3-1, dd3-2). Capillaries (Nanotemper) were filled with 0.5 mg/ml protein samples in three replicates. Melting scan was performed across the temperature range from 20 °C to 90 °C with a temperature ramp of 1 °C/min.

#### Surface plasmon resonance binding assays

Multi-cycle kinetics experiments were performed on a Biacore X100 system (Cytiva). For measuring binding to EGF, EGF (Peprotech) was diluted to 100 μg/mL in 10 mM acetate buffer pH 4.0 and immobilized on the surface of a CM5 sensor chip (Cytiva) using standard amine coupling chemistry. Five different concentrations of the sample solution (nanomolar range) were injected over the functionalized sensor chip surface for 120 s, followed by a 180 s dissociation with the running buffer. At the end of each run, the sensor surface was regenerated with a 30 s injection of 50 mM HCl at a flow rate of 10 μL/min. For measuring binding to HB-EGF, HB-EGF (R&D Systems) was diluted to 20 μg/mL in 10 mM acetate buffer pH 5.0 and immobilized on the surface of a CM5 sensor chip using standard amine coupling chemistry. Five different concentrations of the sample solution (nanomolar range) were injected over the functionalized sensor chip surface for 180 s, followed by a 180 s dissociation with the running buffer. At the end of each run, the sensor surface was regenerated with a 30 s injection of 50 mM NaOH at a flow rate of 10 μL/min. For measuring binding to TGF-α, TGF-α (R&D Systems) was diluted to 100 μg/mL in 10 mM acetate buffer pH 4.5 and immobilized on the surface of a CM5 sensor chip using standard amine coupling chemistry. Five different concentrations of the sample solution (nanomolar – low micromolar range) were injected over the functionalized sensor chip surface for 60 s, followed by a 60 s dissociation with the running buffer. At the end of each run, the sensor surface was regenerated with a 60 s injection of 10 mM glycine-HCl pH 1.5 at a flow rate of 10 μL/min. In all experiments, reference surfaces were treated in the same manner (surface activation and deactivation with amine coupling reagents), except that no ligand was added. Test proteins were diluted in the running buffer (PBS supplemented with 0.05% v/v Tween-20). Analyses were conducted at 25°C at a flow rate of 10 μL/min. The reference responses and zero-concentration sensograms were subtracted from each dataset (double-referencing). Association rate (*k*_*a*_), dissociation rate (*k*_*d*_), and equilibrium dissociation (*K*_*d*_) constants were obtained using the Biacore X100 Evaluation Software. Fitting was performed with global parameter settings where single parameter value applies to the whole titration series. To estimate the reliability of the fit, the fitting procedure was repeated using 4 out of 5 analyte concentrations (excluding one concentration at a time), yielding average values and standard deviations for a single titration series. For binding assays of d3-wt and dd3-2 vs EGF, HB-EGF, and TGF-alpha, two independent titration series were performed.

#### A431 cell proliferation assay

A431 cells were cultured in DMEM medium (Gibco) supplemented with 10 % FBS (Gibco). Cells were pelleted by centrifugation at 300 g for 5 min, washed once with DPBS (Gibco) and once with non-supplemented DMEM medium. After the last washing step, cells were resuspended in DMEM medium supplemented with 1 % FBS and 400 pM EGF (Peprotech). Cell suspension was seeded in a 96-well plate (Corning), 100 μl/well, at a density of 8000 cells/well. Different concentrations of d3-wt (0.032 nM – 500 nM), dd3-2 (0.0064 nM – 100 nM), or cetuximab (0.0064 nM – 100 nM) were added to the wells in triplicates. PBS was added to the wells serving as an untreated control. Several wells contained cells in DMEM medium supplemented with 1 % FBS, but without EGF, as an unstimulated control. After incubation for 72 h at 37°C, 5% CO_2_, 20 μL of CellTiter-Blue® Reagent (Promega) were added to the wells and the plate was incubated for additional 2 h under the same conditions to allow cells to convert resazurin to resorufin. Cell viability was monitored by measuring fluorescence (560/590 nm) using a Synergy HTX Microplate Reader (BioTek). The data were presented as percentage of unstimulated (i.e., without EGF) control fluorescence values.

#### Effect of EGFR inhibitors on zebrafish embryos

Eggs were collected and placed at 28 °C in E3 medium (5 mM NaCl, 0.17 mM KCl, 0.4 mM CaCl_2_, and 0.16 mM MgSO_4_). The age of the embryos and larvae is indicated as hours postfertilization (hpf) or days post fertilization (dpf). All experiments described in the present study were conducted on embryos younger than 5 dpf. To test the effect of the designed inhibitors on zebrafish, we injected around 4 nl of Cetuximab (5.0 mg/ml), dd3-2 (0.2 and 1.0 mg/ml), d3-wt (0.3 and 1.3 mg/ml) into the yolk of embryos at 4-6 hpf and then continued the treatment by adding the inhibitors into medium until 4 dpf. Embryos were distributed in pools of 10-15 into 24-well plates in E3 medium. Survival ratio was assessed every day from 1 dpf to 4 dpf and morphological or developmental defects were analyzed on fixed and stained embryos with Alcian blue at 4 dpf using a Nikon SMZ18 stereomicroscope with a DS-Fi3 camera (5,9 MP). GraphPad Prism software (version 7) was used for graphing and statistical analysis. Cartilage was stained with Alcian Blue 8 GX (Sigma). Zebrafish larvae were fixed in PFA 4 % for 2 h at room temperature, rinsed with PBST and stained overnight with 10 mM MgCl_2_/80 % ethanol/0.04 % Alcian Blue solution. Embryos were rinsed with 80 % ethanol/10 mM MgCl_2_ and washed stepwise with 70 %, 50 %, 30 % ethanol and PBST. Pigments were bleached in H_2_O_2_ 3 %/formamide 5 %/20X SSC 2,5 % up to 30 minutes. Embryos were stored in 80 % glycerol for imaging.

#### Design of copper-binding proteins

Combinatorial design simulations were run to redesign the template structure of apo-protein form (PDB: 5FJD), given the limitations of the described method and the used classical mechanics force field to adequately describe coordinated metal ions. Most surface positions were set as designable to break the oligomerization interfaces and stabilize the helical structures. The spec file (Methods S1) was run 100 instances, for 125 CPU hours/instance. The final sequences were chosen according to the same aMD filtering described above. Through a second round of computational design, we sought to create constructs with a more sealed core. This was done by mutating 3 or 6 cysteine residues (and their surrounding positions) into well-packed hydrophobic residues at the end of the cysteine-lined lumen of the plr1 model. Three such design candidates were also synthesized and tested: cr3, cr61, and cr62. Additionally, the surface positions of plr1 were also redesigned to bias them towards negative supercharged variants, where two candidates were selected and tested: neg1 and neg2.

#### Purification of copper-binding proteins

The synthetic genes for all tested designs, and the design template (Table S2) were cloned without purification tags in a pET28b(+) vector (BioCat GmbH). The proteins were transformed and expressed in *E. coli* BL21 (DE3). Expression was induced in 2-litre LB medium supplemented with 40 μg/ml kanamycin at an optical density (OD600) of 0.8, and was done overnight at 25 °C. Cells were harvested by centrifugation at 5000 g at 4 °C for 15 min and lysed in 30 ml of lysis buffer (for positively charged variants: 100 mM NaCl, 50 mM Tris-HCl pH 8.0; for negatively charged variants: 2 mM EDTA, 20 mM Tris-HCl pH 8.0) supplemented with a tablet of the cOmplete, EDTA-free Protease Inhibitor Cocktail (Roche) and 3 mg of lyophilized DNase I (PanReac AppliChem) using a Branson Sonifier 250 (Fisher Scientific). The lysate was cleared by centrifugation at 28000 g at 4°C for 50 min and the supernatant was filtered through a 0.45 μm filter (Millipore). The sample was diluted 5-fold and applied to a 5 ml HiTrap Capto Q or S columns depending on their isoelectric point (Cytiva). Positively charged proteins were eluted in 20 mM HEPES, 1 mM DTT buffer pH 7.4, using a gradient of 0 to 1.5 M KCl. Negatively charged variants where eluted in 20 mM HEPES, 1 mM DTT buffer pH 8.0, using a gradient of 0 to 1.5 M NaCl. The relevant fractions were identified by SDS-PAGE analysis, and further purified on a Superdex 75 Increase 10/300 GL gel filtration column (Cytiva) using 20 mM HEPES, 150 mM NaCl buffer pH 7.4. Gel filtration fractions containing pure protein in the desired oligomeric state were pooled, concentrated, and stored at -20°C for subsequent analyses.

#### Thermostability analysis of copper binders

NanoDSF measurements using Prometheus NT.48 (Nanotemper) were performed to evaluate thermostability of selected designs, as well as thermostability of Csp1. Capillaries (Nanotemper) were filled with 0.1-1 mg/ml protein samples in 3 replicates. Melting scan was performed across the temperature range from 20 °C to 110 °C with a temperature ramp of 1 °C/min. In addition to measuring the intrinsic fluorescence intensity ratio (350/330 nm), light intensity loss due to scattering (backreflection) was measured to detect protein aggregation.

#### Cu^2+^-binding affinity, capacity, and stability

To evaluate how many Cu^2+^ ions can be bound within the core of plr1, absorption spectra (from 400 nM to 700 nM) were recorded for samples containing 20 μM of Zincon (Supelco), 20 μM of CuSO_4_ and varying concentrations of plr1 (to provide ratio Cu^2+^/plr1 from 1 to 19). In case, when plr1 is saturated with Cu^2+^ ions, complex between Cu^2+^ and Zincon forms and characteristic Cu^2+^ZI peak at 599 nM can be observed on the spectrum.

The binding affinities of the designs to Cu^2+^ were determined using a modified Zincon assay described by Kocyla et al.^57^ Zincon competition tests with plr1 and neg1 were performed in 20 mM HEPES buffer, pH 7.4, containing 150 mM NaCl. 50 μM of Zincon was mixed with 20 μM of CuSO_4_ and different concentrations of a protein solution (0 – 160 μM for plr1, 0 – 80 μM for neg1). Samples were incubated for 8 h at 25 °C. The exact concentrations of Cu^2+^ZI complex present in each sample were calculated based on the absorbances at 599 nM using the molar absorption coefficient of Cu^2+^ZI at pH 7.4, 26100 M^-1^cm^-1^. Absorbances of the samples were measured on a Synergy HTX Microplate Reader (BioTek) in a 384-well plate (Greiner). The dissociation constant of the designed proteins ($K_{d}^{Des}$) was calculated as follows:

(Equation 8) $$K_{d}^{Des}=\frac{K_{d}^{{Cu}^{2+}ZI}}{K_{ex}}$$

where $K_{d}^{{Cu}^{2+}ZI}$ is a dissociation constant of Cu^2+^ZI at pH 7.4, 4.68×10^-17^ M, and $K_{ex}$ is a constant describing the reaction of Cu^2+^ ion transfer from Cu^2+^ZI to the tested copper-binding design. $K_{ex}$ was determined by fitting the experimental data to the following equation:

(Equation 9) $$K_{ex}=\frac{\left[ZI\right]\left[{Cu}^{2+}Des\right]}{\left[{Cu}^{2+}ZI\right]\left[Des\right]}$$

To measure off rates for Cu^2+^ dissociating from plr1 or neg1, we performed the dissociation experiments in 20 mM HEPES buffer, pH 7.4, containing 150 mM NaCl and 25 % v/v of untreated fetal bovine serum. Samples containing 50 μM of Zincon, 50 μM of CuSO_4_ and 150 μM of either plr1 or neg1 were incubated in 384-well plate (Greiner) for 48 h at 37 °C. Every hour the absorbances at 599 nM were recorded. Samples containing no Cu^2+^ and no protein were used as a control, and average absorbance of these samples was subtracted from all tested values. Results are presented as a decrease in amount of Cu^2+^ bound (${Cu}^{bound}$) to the protein over time (*t*). Absorbance values from the wells containing Zincon and Cu^2+^, but no protein were used for normalization and were referred to as 100 % of unbound Cu^2+^. Dissociation rates ($K_{off}$) were determined by fitting the experimental data to the equation:

(Equation 10) $${Cu}^{bound}={Cu}_{0}^{bound}\times e^{-K_{off}\times t}$$

where ${Cu}_{0}^{bound}$ is a percent of Cu^2+^ bound to the protein at time zero.

#### Loading of copper binders with ^64^Cu

^64^Ni (98 % enrichment) was electroplated on a Pt/Ir plate (90/10) and irradiated with 12.5 MeV protons on the Tübingen PETtrace cyclotron (GE Healthcare) to produce ^64^Cu via the ^64^Ni(p,n)^64^Cu route. The target was dissolved using concentrated HCl and ^64^Cu^2+^ was purified using ion chromatography as described before.^58^ The obtained radioisotope solution in 0.1 M HCl was buffered with 1.5 volumes of 0.5 M ammonium acetate pH 4.1 before addition of the protein (2 μg per MBq). After 30 min of incubation at 35 °C incorporation of the radioactivity was analyzed by thin layer chromatography (stationary phase: Polygram SIL G UV254, Macherey-Nagel; mobile phase: 0.1 M sodium citrate pH 5) with autoradiographic detection using a phosphor imager (Cyclone Plus Storage Phosphor System, Perkin Elmer). Size exclusion chromatography with radioactivity detector (1260 Infinity II, Agilent; Superdex 75 Increase 10/300, Cytiva) was used to analyze the elution profile of the protein and bound radioactivity. For stripping of the bound radioactivity DTPA (final concentration 0.14 mg/ml) was added to the labeled protein and re-analyzed after incubation at room temperature.

### Quantification and statistical analysis

To evaluate scoring accuracy of the Damietta potential against different stability benchmarks the Pearson correlation coefficients and the correlation p-values were calculated using the scipy.stats sub-package in the SciPy (version 1.8.0). For SPR measurements, A431 cell proliferation, as well as copper-binding experiments, mean values and standard deviations were calculated and described in the Figure legends. Statistical analysis for zebrafish experiment was performed using the GraphPad Prism software (version 7).

## Data Availability

• This paper analyzes existing, publicly available data. These accession numbers for the datasets are listed in the key resources table. • Damietta software is available at https://bio.mpg.de/damietta. An archival DOI is listed in the key resources table. • Any additional information required to reanalyze the data reported in this paper is available from the lead contact upon request.

## Supplemental information

Document S1. Figures S1–S10, Tables S1–S3, and Methods S1

Document S2. Article plus supplemental information
