# Supplementary material for: The design of functional proteins using tensorized energy calculations
Source: Cell Rep Methods. 2023 Aug 15;3(8):100560. doi: 10.1016/j.crmeth.2023.100560 (PMC10475850; doi:10.1016/j.crmeth.2023.100560)
Supplement: Document S1. Figures S1–S10, Tables S1–S3, and Methods S1 [file mmc1.pdf]

Cell Reports Methods, Volume *3*

Supplemental information

The design of functional proteins

using tensorized energy calculations

Kateryna Maksymenko, Andreas Maurer, Narges Aghaallaei, Caroline Barry, Natalia Borbarán-Bravo, Timo Ullrich, Tjeerd M.H. Dijkstra, Birte Hernandez Alvarez, Patrick Müller, Andrei N. Lupas, Julia Skokowa, and Mohammad ElGamacy

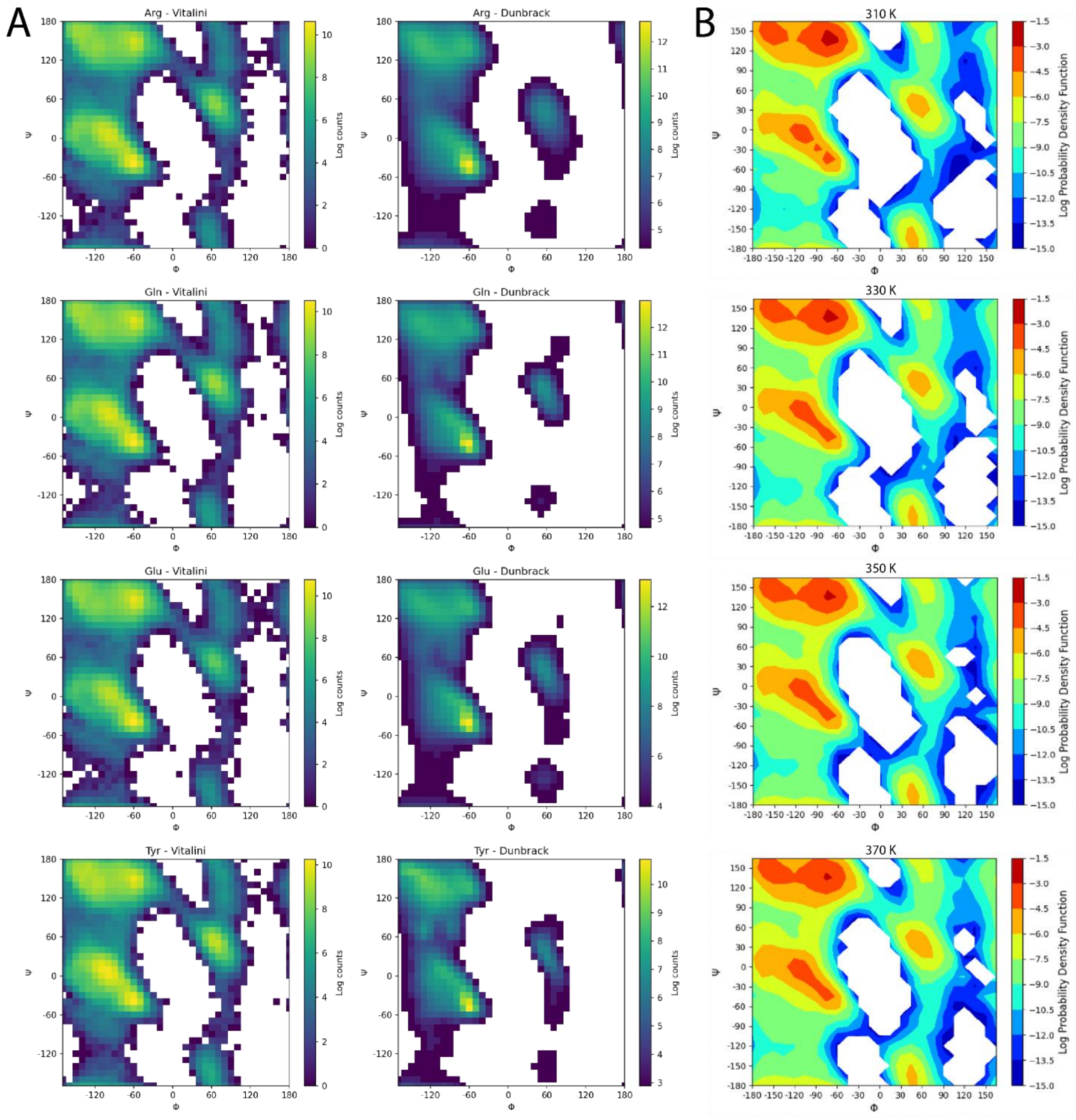

Supplemental figure 1: MD-derived conformational distributions cover broad conformational space, related to STAR Methods. (A) The figure shows conformational distributions for four amino acid examples with diverse physical properties (Arg, Gln, Glu, and Tyr; top to bottom). Left column shows distributions used to drive the described rotamer library (originally described by Vitalini et al. [S1]). Right column shows PDB-derived distributions described by Shapovalov and Dunbrack [S2]. **(B)** MD performed at elevated temperatures can be used to obtain broadened conformational distributions to better cover rare conformations. Long MD simulations (1 μs) of capped GAG tripeptide yield broader coverage of the alanine (φ, ψ)-space at successively higher temperatures of 310 K, 330 K, 350 K, 370 K, without perturbation of the overall distribution.

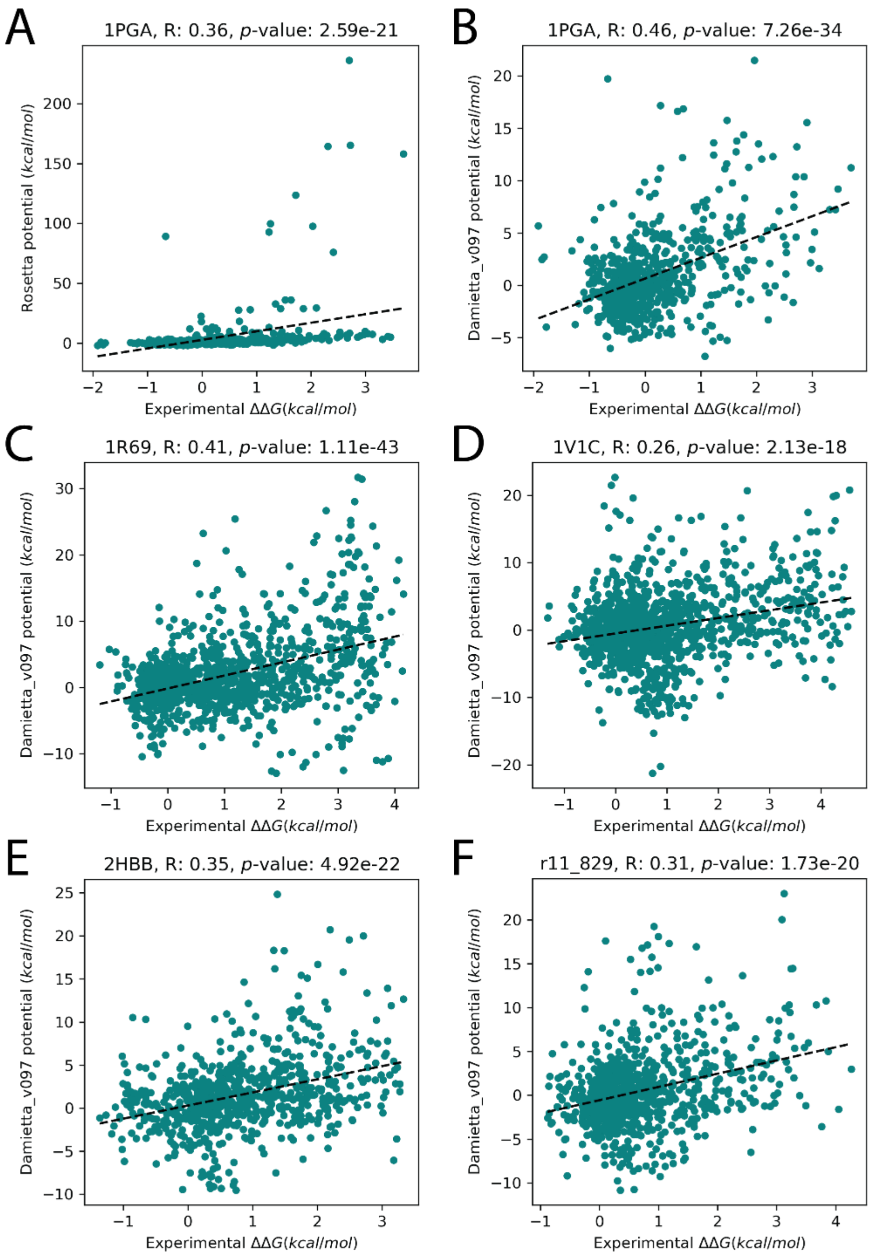

Supplemental figure 2: Retrospective validation of the Damietta potential against five different benchmarks, related to Fig 1. A comparison between the Rosetta **(A)** and Damietta **(B)** energies correlation with the change in folding free energy (ΔΔG) values for mutants of Gβ1 protein. Evaluation of the Damietta energy function performance against a benchmark of mutants of the N-terminal domain of phage 434 repressor (PDB 1R69) **(C)**, SH3 domain in human obscurin (PDB 1V1C) **(D)**, N-terminal domain of ribosomal protein L9 (PDB 2HBB) **(E)**, and the hallucination design r11_829_TrROS **(F)**. The Pearson correlation coefficients (R) and the correlation p-values are shown.

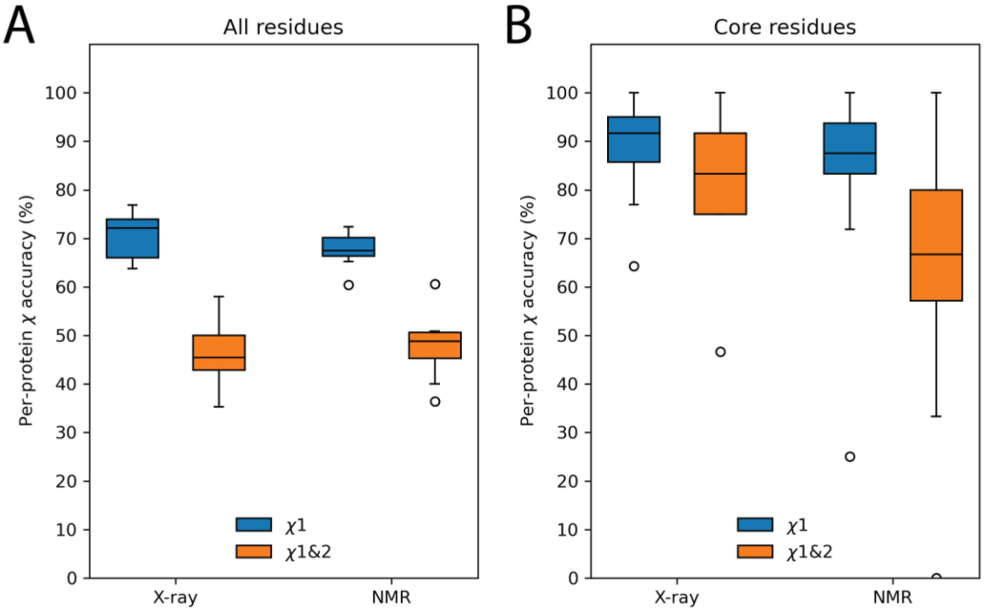

C

PDB	1TTZ	1TVG	2O0Q	2Q00	3C4S	3FIF	3H9X	3IDU	3K63
Sequence recovery (%)	12.33	11.94	14.41	10.92	12.73	18.75	11.30	11.43	9.52

Supplemental figure 3: Results of native side-chain conformation recovery and single-position sequence recovery, related to Fig. 1. (A) Boxplot shows the median χ_1 prediction accuracy to be around 70 % for both X-ray and NMR structures. $\chi_{1\&2}$ accuracies are roughly 20 % lower than χ_1 accuracies. **(B)** Rotamer recovery for core positions was found to be high with the median χ_1 accuracy of around 90 % and the median $\chi_{1\&2}$ accuracy of around 65-85 %. **(C)** Sequence recovery rates for 9 different crystal structures are presented as a percentage of amino acid positions within a protein at which the lowest-energy residue selected by `sp` sampler is identical to the native amino acid.

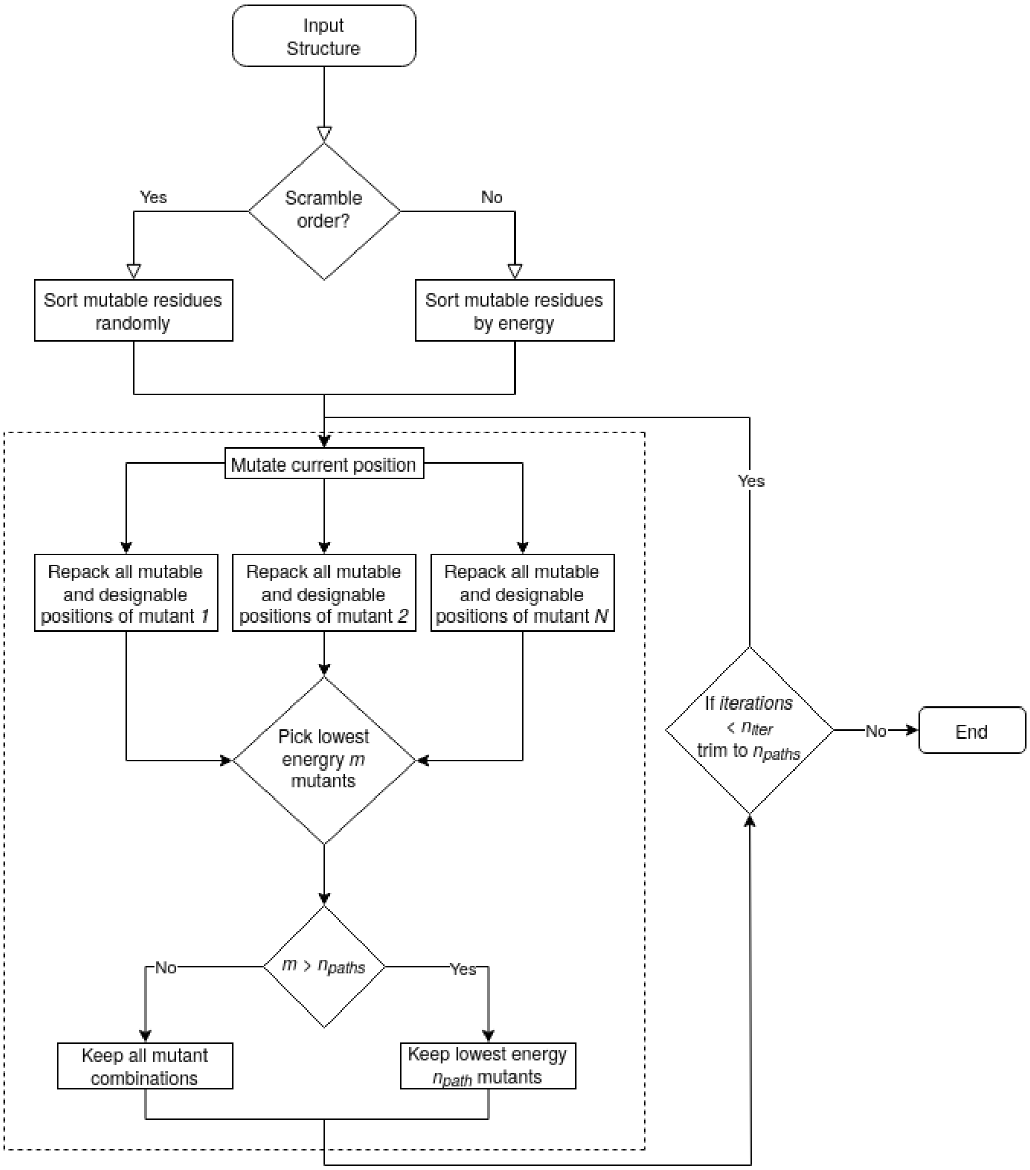

Supplemental figure 4: A flow chart of the greedy branch-and-rank combinatorial sampling algorithm used for design, related to Fig. 1. Scheme of the few-to-many-to-few combinatorial sampler. The algorithm assumes all mutable and repackable residues are inter-dependent and follows a maximum number of paths n_{paths} down the decision tree. At the start of the design simulation all of the combinatorially generated sequences are further designs so long as their number is $\leq n_{paths}$. But as the sequence combinations grow $> n_{paths}$, the algorithm ranks all of the designs by the average energy per residue (evaluated at the mutable and repackable positions). Only a small number ($= n_{paths}$) of lowest energy mutants is kept for the next position mutagenesis round. The order of mutable positions can be preset or randomized as the defined by the user. The main flow of sampling therefore follows: i) mutate position, ii) calculate average energy of every mutant at that position, iii) combine the top m mutations with all of the previously kept mutants from the previous cycle to evaluate the average energy per residue, iv) if the generated combinations are larger in number than n_{paths}, only keep the lowest energy n_{paths} sequences, v) move to the next mutable position and repeat step, vi) repeat these iterations over all mutable residues. This cycle is repeated n_{iters} times for better convergence.

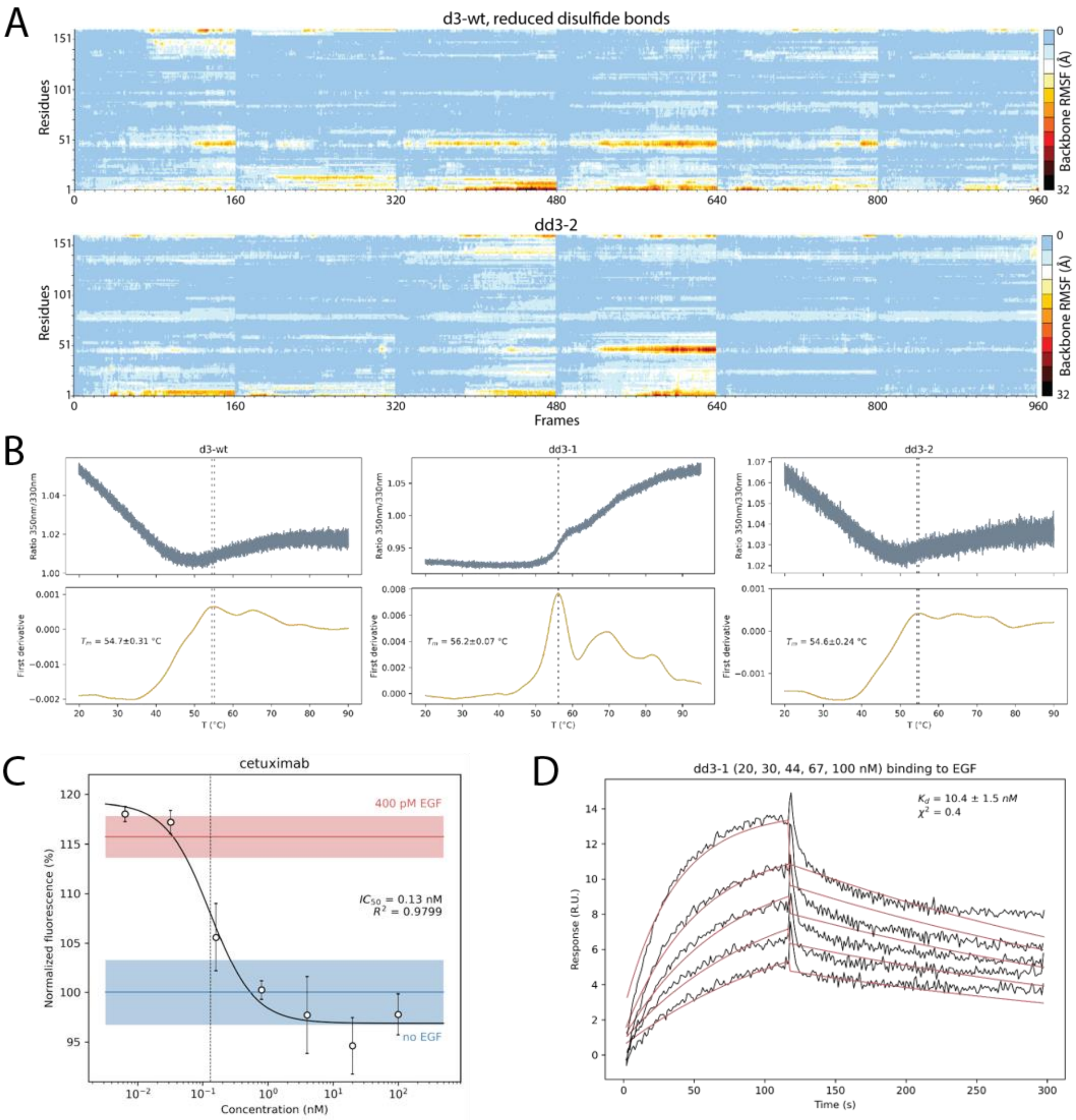

Supplemental figure 5: Computational and experimental characterization of EGFR inhibitors, related to Fig. 2. (A) RMSF heatmaps indicate improved conformational stability of dd3-2 (bottom) compared to the reduced cysteines form of d3-wt (top). A single data point in the heatmap represents RMSF for each residue's backbone atoms and its reference residue's backbones atoms (frame 0) through tempering MD trajectories (160 frames, 6 replicas). **(B)** NanoDSF measurements showed no significant difference in melting temperatures between designed proteins (dd3-1, dd3-2) and the wild type (d3-wt). Melting temperatures (T_m) are represented as mean ± SD. **(C)** EGFR-blocking antibody Cetuximab inhibited proliferation of A431 cells with IC_{50} of 0.13 nM, which is only 3-fold lower compared to IC_{50} of dd3-2 (Fig. 2C). The positive and negative control values of cell proliferation with and without EGF-treatment are indicated by red and blue lines, respectively. Shades and error bars represent the standard deviation across three replicates. **(D)** SPR sensograms showed dd3-1 design to bind EGF with K_d value of 10 nM, which is approximately 6-fold tighter compared to d3-wt (Fig. 2B). K_d is represented as mean ± standard deviation (SD). Experimental data, black; fit, red.

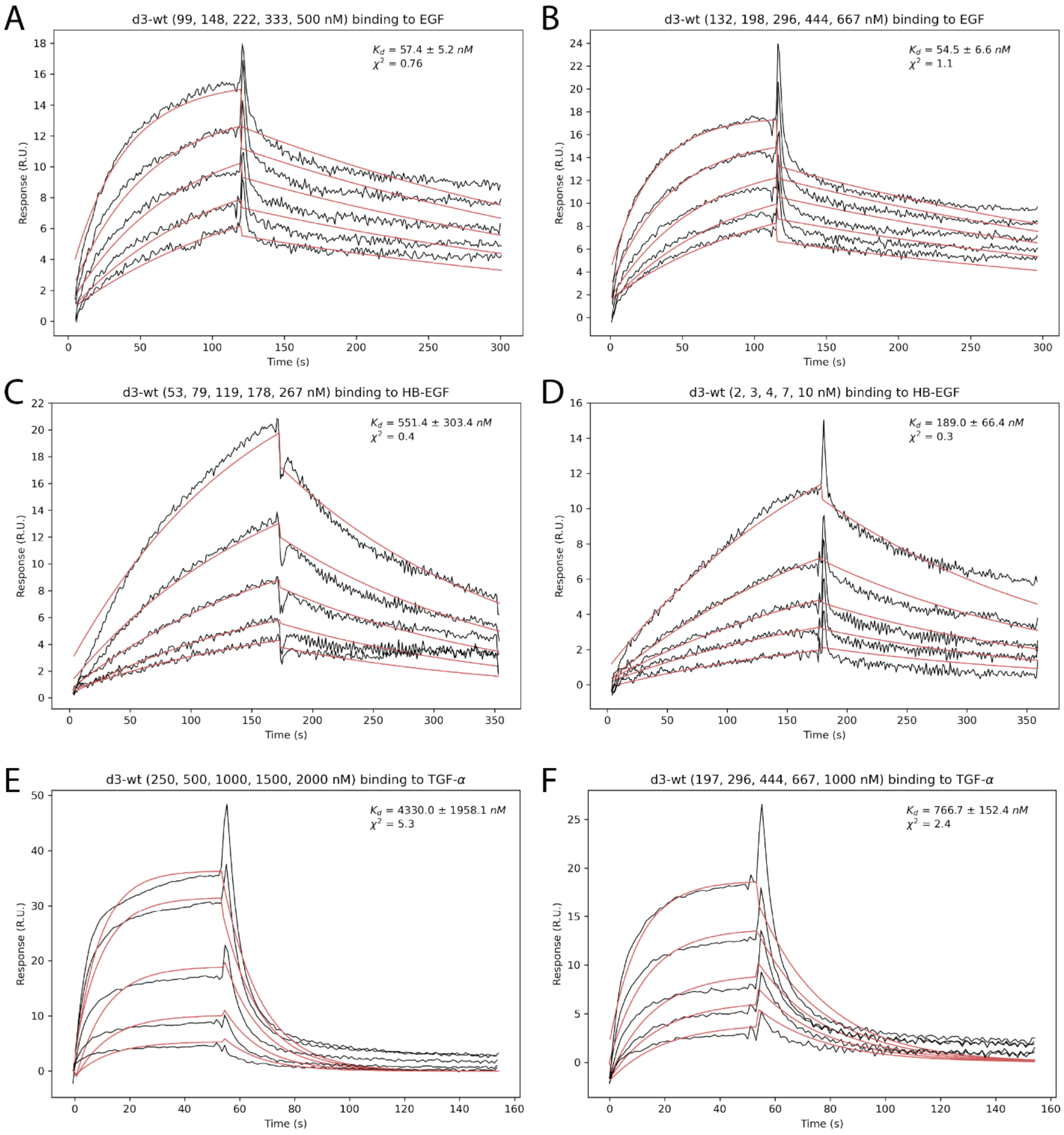

Supplemental figure 6: SPR titrations of d3-wt binding to immobilized EGFR ligands, related to Table 1. Sensograms of d3-wt binding to **(A, B)** EGF, **(C, D)** HB-EGF, and **(E, F)** TGF-alpha. Results were obtained from two independent experiments. K_d is represented as mean ± standard deviation (SD). Experimental data, black; fit, red.

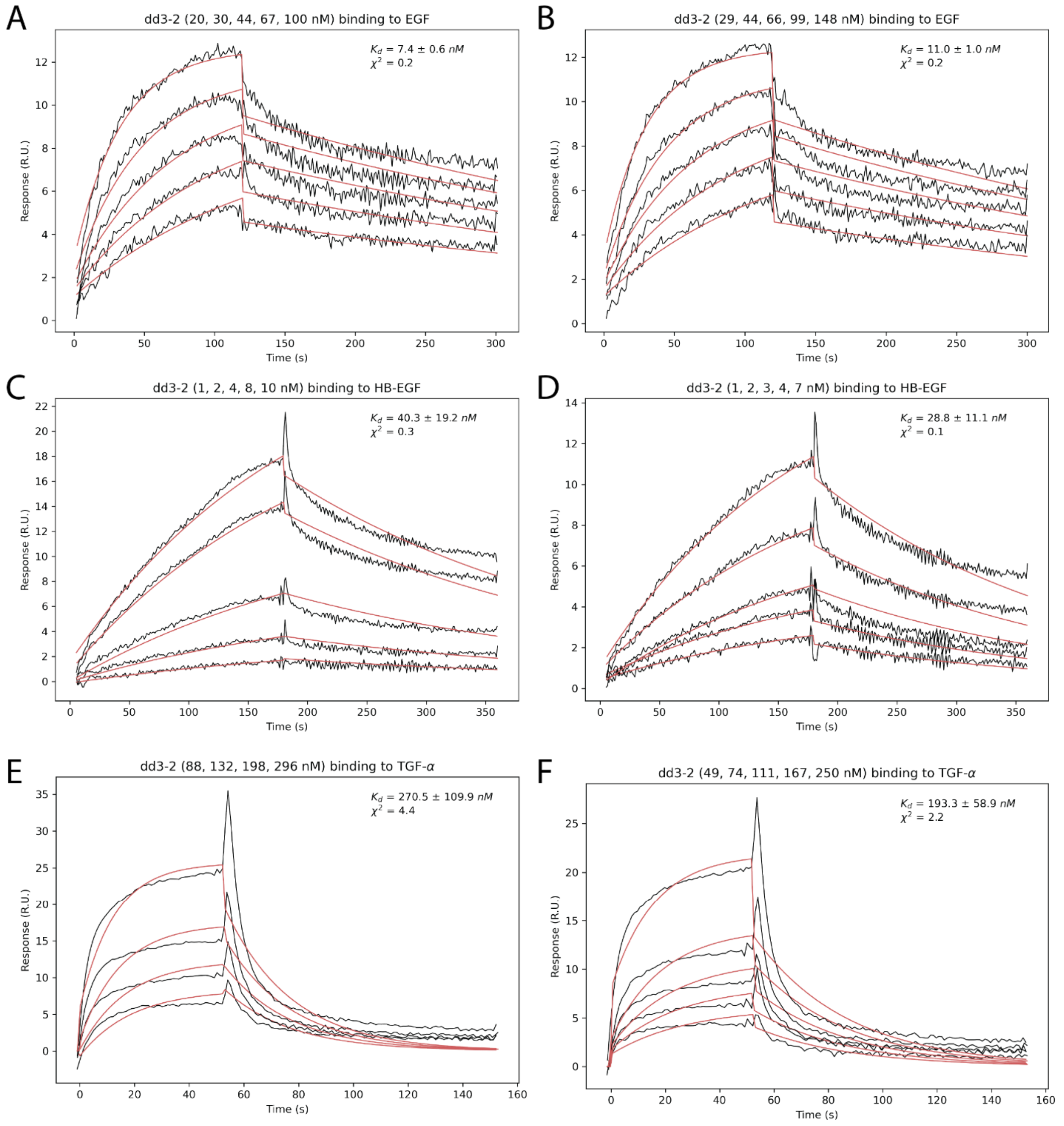

Supplemental figure 7: SPR titrations of dd3-2 binding to immobilized EGFR ligands, related to Table 1. Sensograms of dd3-2 binding to **(A, B)** EGF, **(C, D)** HB-EGF, and **(E, F)** TGF-alpha. Results were obtained from two independent experiments. K_d is represented as mean ± standard deviation (SD). Experimental data, black; fit, red.

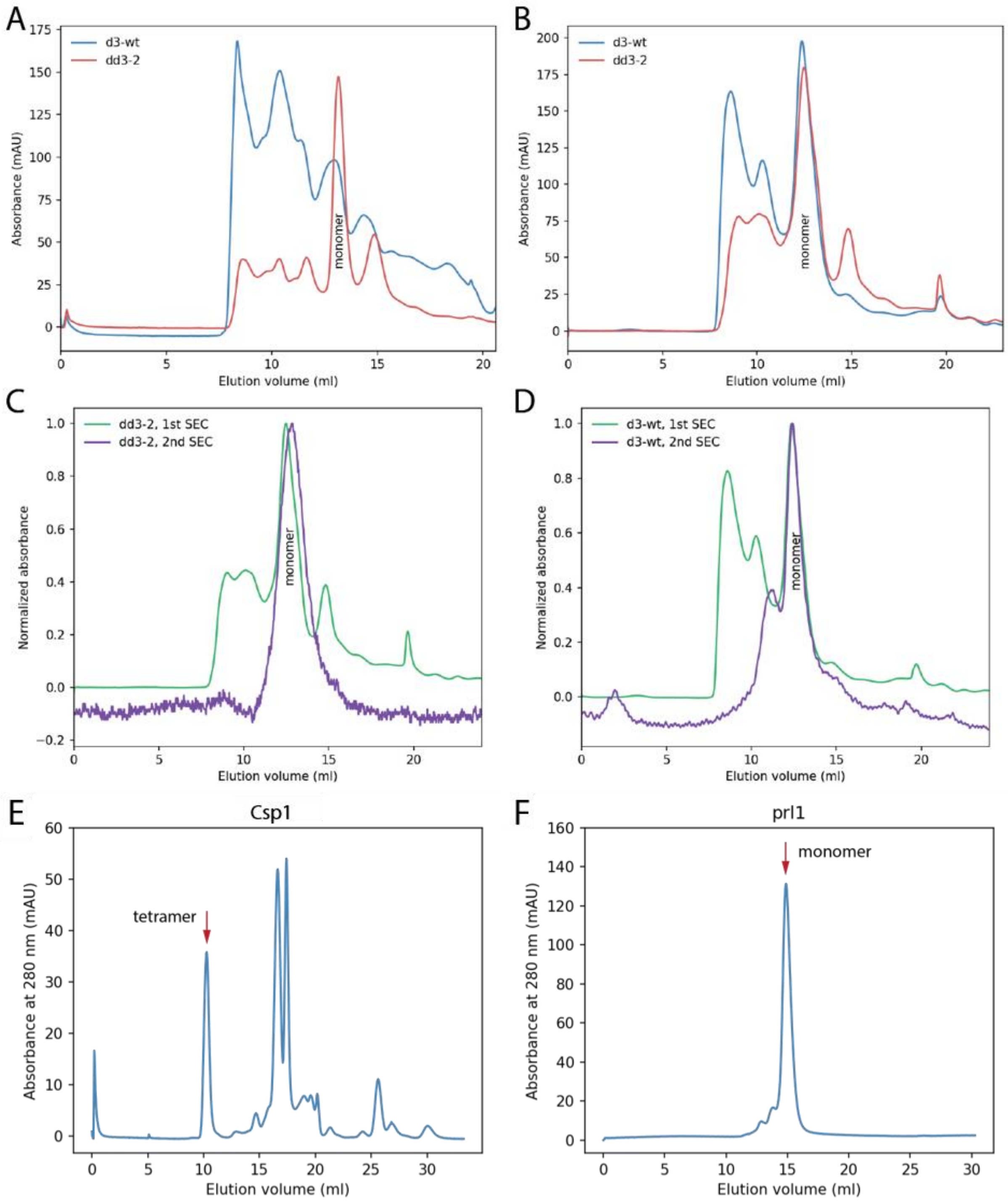

Supplemental figure 8: Analytical size-exclusion profiles of EGFR-inhibiting and copper-binding proteins, related to Fig. 2 and Fig. 4A. (A, B) The chromatograms for d3-wt and dd3-2 proteins, obtained from two independent rounds of expression and purification, show d3-wt to have higher aggregation propensity than dd3-2. For experimental evaluation, monomeric fractions of dd3-2 and d3-wt were collected. **(C)** Analytical size-exclusion elution profile of the collected dd3-2 monomeric fraction. **(D)** Analytical size-exclusion elution profile of the collected d3-wt monomeric fraction. **(E)** Csp1 protein elutes as a tetramer and forms aggregates in solution. **(F)** plr1 elutes as a homogenous monomer with no signs of aggregation.

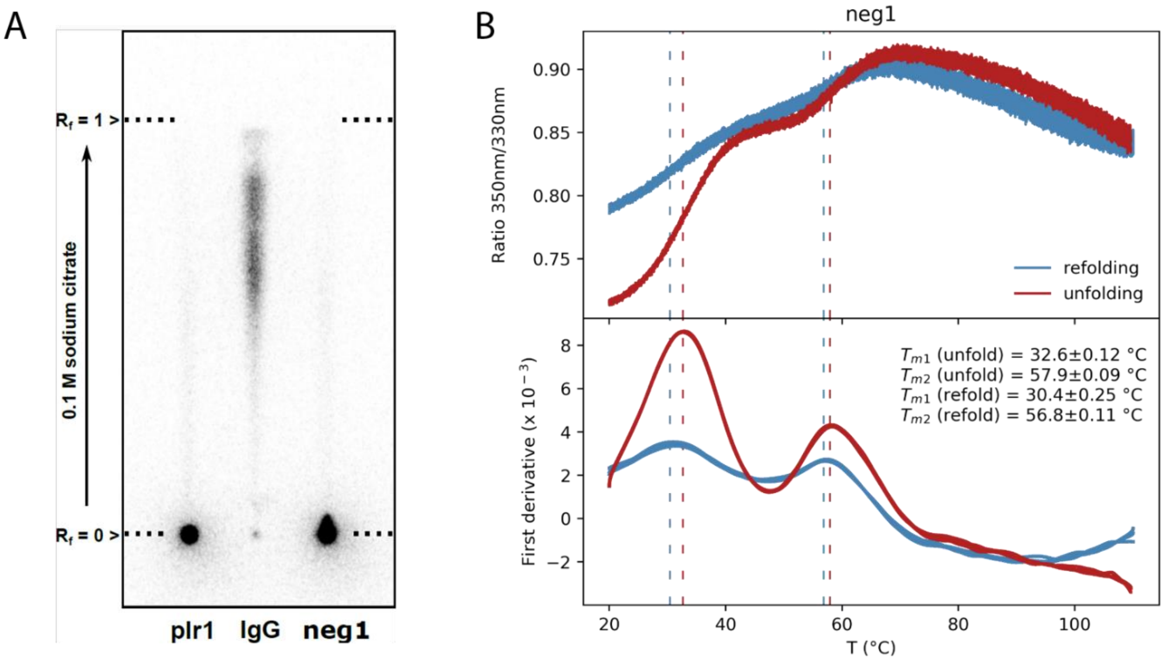

Supplemental figure 9: Experimental characterization of copper-binding designs, related to Fig. 3. **(A)** Radio-TLC shows the specific binding of plr1 or neg1, but not IgG (negative control) to $^{64}Cu^{2+}$ ions. **(B)** NanoDSF measurement shows substantial destabilization of the negatively supercharged variant neg1 when compared to plr1 (Fig. 3B). Specifically, two melting transitions T_{m1} and T_{m2} occur at around 33 °C and 58 °C, respectively (red lines show differential fluorescence data points and first derivatives of three replicas of the heating ramp). These two unfolding events however appear to be reversible (blue lines show the behavior down the cooling ramp). Melting temperatures (T_m) are represented as mean ± SD.

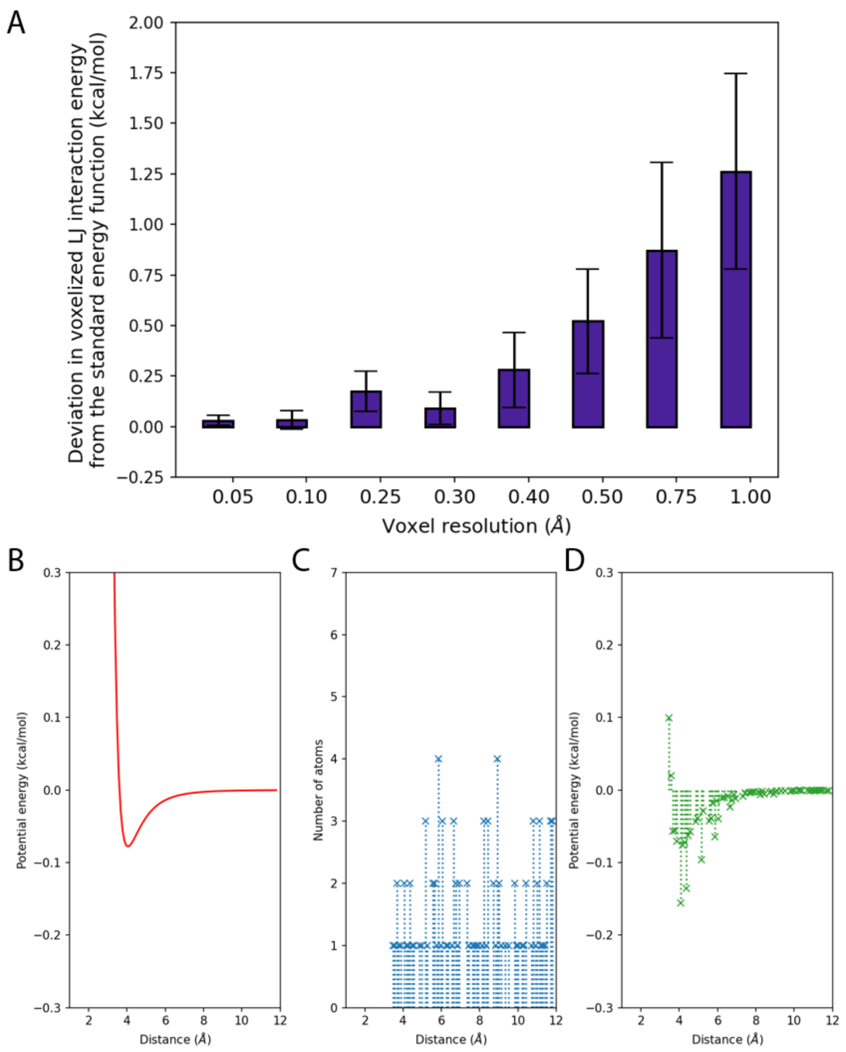

Supplemental figure 10: Array-oriented calculation of interactions and the effect of voxel resolution on energy error, related to STAR Methods. (A) The Y-axis shows the residual energy values by subtracting energy values calculated by the standard LJ function from the discretized LJ function; i.e. $E_{LJ,discretized} - E_{LJ,smooth}$. The discretization was performed at voxel resolutions of 0.05, 0.10, 0.25, 0.30, 0.40, 0.50, 0.75 and 1.00 Å. In this demonstration, the interactions between one sp^3 methyl carbon ($\varepsilon_{LJ} = -0.078\,kcal/mol$, $\sigma_{LJ} = 3.63$ Å) and 100 inbound sp^3 methyl carbons, that are randomly pooled from a uniform distribution, bounded at the distance range of 3.5 to 12.0 Å. Error bars represent standard deviation values for 10 replicas, with randomized atomic positions. **(B-D)** The presented framework simplifies interection calculations to an expensive pre-computed field of a discrete rotamer (**B**; here shown as a 1D LJ potential of a single atom for simplicity) and a quickly populated histogram of the environment atomic positions (**C**; here depicted as 1D array for simplicity). A single multiplication process yields the “energies array” **(D)**.

Table S1: The time performance of the combinatorial sampler under different simulation parameters, related to Fig. 1. These design simulations were run on the ubiquitin crystal structure (PDB: 1UBQ) as template.

	Simulation 1	Simulation 2	Simulation 3	Simulation 4	Simulation 5
Mutable residues	10	20	30	40	40
Repackable residues	10				
Target mutations per position	20				
Conformers per repacking task	100				
Number of iterations (`n_iters`)	1				
Theoretical sequence complexity	$1 \cdot 10^{13}$	$1 \cdot 10^{26}$	$1.1 \cdot 10^{39}$	$1.1 \cdot 10^{52}$	$1.1 \cdot 10^{52}$
Theoretical rotameric complexity	$1 \cdot 10^{53}$	$1 \cdot 10^{86}$	$1.1 \cdot 10^{119}$	$1.1 \cdot 10^{152}$	$1.1 \cdot 10^{152}$
Intermediate mutants (`m_muts`)	3				
swarm paths (`n_paths`)	6				1
Actual rotamer evaluations	$1.4 \cdot 10^{7}$	$9.2 \cdot 10^{7}$	$3.3 \cdot 10^{8}$	$6.4 \cdot 10^{8}$	$1.9 \cdot 10^{8}$
Wall-clock time (s)	$1.3 \cdot 10^{3}$	$3.5 \cdot 10^{3}$	$7.3 \cdot 10^{3}$	$1.1 \cdot 10^{4}$	$3.7 \cdot 10^{3}$
CPU threads	8 (2.3 GHz)				
Time/residue* (s)	$9.3 \cdot 10^{-3}$	$3.8 \cdot 10^{-3}$	$2.2 \cdot 10^{-3}$	$1.7 \cdot 10^{-3}$	$1.9 \cdot 10^{-3}$
Time/rotamer (s)	$9.3 \cdot 10^{-5}$	$3.8 \cdot 10^{-5}$	$2.2 \cdot 10^{-5}$	$1.7 \cdot 10^{-5}$	$1.9 \cdot 10^{-5}$
Time/atom-step**	$8.5 \cdot 10^{-8}$	$3.5 \cdot 10^{-8}$	$2.0 \cdot 10^{-8}$	$1.6 \cdot 10^{-8}$	$1.7 \cdot 10^{-8}$
Lowest-energy decoy (kcal/mol)***	-11.0	-11.9	-12.3	-12.2	-11.9

* Evaluating 100 rotamers per residue ranged between 0.002 ~ 0.009 s. This is in comparison with ~ 0.2 s for RaSP, 100 ~ 200 s for Rosetta, 200 ~ 400 s for FoldX, 0.7 ~ 0.8 s for ACDC-NN, or 4 ~ 6 s for ThermoNet [S3].

** Assuming 9.7 $Å^3$/atom as an average atomic volume occupancy per simulation cube (1098 atoms / 10,648 $Å^3$). The tabulated results describe the overall performance across the entire control flow of the `cs_f2m2f` application. For instance, the isolated speed of the LJ energy function, on a single CPU core gives 130 ns/atom-step (including environment mapping and rotamer·environment tensor multiplication), which is substantially faster than recently described approaches [S4].

*** As the average energy per sampled residues (i.e. all repackable or mutable residues).

Table S2: Protein sequences of template and designed EGFR inhibitors and copper-binding proteins, related to STAR Methods. Mutated residues are highlighted in yellow.

Name	Sequence
**d3-wt**	CNGIGIGEFKDSLSINATNIKHFKNCTSISGDLHILPVAFRGDSFTHTPPLDPQELDILKTVKEITGFLLI<br>QAWPENRTDLHAFENLEIIRGRTKQHGQFSLAVVSLNITSLGLRSLKEISDGDVIISGNKNLCYANTINWK<br>KLFGTSGQKTKIISNRGENSCKATGQ
**dd3-1**	ANGIGIGEFKDSLSIWAWNIKHFKNARSISGDLHILPVAFRGDSFTHTPPLEPKELEILKTVKEITGYLLV<br>QAWPENRTDLHAFENLEIIRGRTKQHGQFSLAVVSLNVTSLGLRSLKEISDGDVIMSGNKNLKMANEMNWK<br>KMFGTSGQKTKIISNRGE--------
**dd3-2**	ANGIGIGEFKDSLSINAWNIKHFKNAQSISGDLHILPVAFRGDSFTHMPPLEPKELEILKTVKEITGYLLI<br>QAWPENRTDLHAFENLEIIRGRTKQHGQFSLAVVSLNVTSLGLRSLKEISDGDVIMSGNKNLKWANKWNWK<br>KVFGTSGQKTKIISNRGE--------
**Csp1**	M--GAKYKALLDSSSHCVAVGEDCLRHCFEMLAMNDASMGACTKATYDLVAACGALAKLAGTNSAFTP<br>AFAKVVADVCAACKKECDKFPSIAECKACGEACQACAEECHKVA
**plr1**	M--GAKYKALLESSRRCVRVGERCLRHCREMLRRNDASMGACTKATYDLVKACAELAKLAGTNSARTP<br>KKAKQVARVCEKCKKECDKFPSIAECKACAEACKKCAEECRKVA
**plr2**	M--GAKYKALLRSSRRCVKVGEECLRHCREMLKRNDASMGACTKATYDLVKACARLAKLAGTNSARTP<br>RRAKRVARVCERCKKECDKFPSIAECKACAEACQRCAEECKKVA
**cr3**	MGHGAKYKALLESSRRCVRVGERCLRHAREMLRRNDASMGALTKATYDLVKACAELAKLAGTNSARTP<br>KKAKQVARVCEKCKKECDKWPSMAEAKACAEACKKCAEECRKVA
**cr61**	MGHGAKYKALLESSRRCVRVGERALRHAREMLRRNDASMGAATKAFYDLVKACAELAKLAGTNSARTP<br>KKAKQVARVCEKCKKEADKWPSYAEAKAAAEACKKCAEECRKVA
**cr62**	MGHGAKYKALLESSRRCVRVGERWLRHAREMLRRNDASMGAATKAAYDLVKACAELAKLAGTNSARTP<br>KKAKQVARVCEKCKKEADKWPSMAEAKAAAEACKKCAEECRKVA
**neg1**	MGHGAHYAALLESSERCVEVGERCLEHCQEMLEKNDESMGACTKATEDLVKACEELAKLAGTESAQTP<br>ELAAEVARVCEQCQKECDKFPSIEECKECAEACQECAEECEKVA
**neg2**	MGHGAHYEALLESSERCVEVGERCLEHCQEMLEKNDESMGACTKATEDLVKACEELAKLAGTESAQTP<br>ELAAEVARVCRQCAKECDKFPSIEECKECAEACEECAEECRKVA

Table S3: Survival of zebrafish embryos exposed to different concentrations of inhibitors, related to Fig. 2D.

Inhibitor	Concentration	Lethality (%)
Cetuximab	5.0 mg/ml (34 µM)	30
d3-wt	0.3 mg/ml (16 µM)	10
	1.3 mg/ml (70 µM)	23
dd3-2	0.2 mg/ml (11 µM)	20
	1.0 mg/ml (55 µM)	48

Supplemental Methods S1: Spec files for d3-wt and Csp1 redesign, related to STAR Methods

d3-wt redesign

```
library                         /damietta_v022/libv021_100
input                           d3wt_autopsf.pdb

# designable residues
mut_res                 1                       AFILMVWY
mut_res                 16                      DNHILMWYF
mut_res                 18                      ANSTQNHILMVWYF
mut_res                 26                      AFILMVWY
mut_res                 27                      STEDNQR
mut_res                 48                      TFILMVWY
mut_res                 52                      DE
mut_res                 54                      QREK
mut_res                 57                      DE
mut_res                 68                      HIFWY
mut_res                 71                      ILV
mut_res                 109                     ILVM
mut_res                 127                     ILMV
mut_res                 134                     DKRQNSTEH
mut_res                 135                     ADEFHIKLMNQRSTVWY
mut_res                 138                     DKRQNSTEH
mut_res                 139                     FILMVWY
mut_res                 144                     FILMVWY

# repacking residues
rpk_res                 23
rpk_res                 29
rpk_res                 46
rpk_res                 55
rpk_res                 60
rpk_res                 69
rpk_res                 78
rpk_res                 81
rpk_res                 96
rpk_res                 104
rpk_res                 107
rpk_res                 112
rpk_res                 117
rpk_res                 125
rpk_res                 130
rpk_res                 133
rpk_res                 154
rpk_res                 157

# sampling parameters (optional)
scramble_order                  1
m_mutations                     3
n_paths                         7
n_iters             5 # default:= 1

# mutagenesis scoring weights (optional)
mut_max_lj                      25.0
mut_w_pp                        1.0
mut_w_k                         0.0
mut_w_lj                        1.0
mut_w_solv                      1.0
mut_w_elec                      0.125

# repacking scoring weights (optional)
rpk_max_lj              25.0
rpk_w_pp                1.0
rpk_w_k                 1.0
rpk_w_lj                1.0
rpk_w_solv              1.0
rpk_w_elec              0.125
```

Csp1 redesign

```
library                         /damietta_v022/libv021_100
input                           cu3_wt_autopsf.pdb

# designable residues
mut_res                 21                      KREQ
mut_res                 24                      KREQ
mut_res                 25                      KREQ
mut_res                 28                      KREQ
mut_res                 32                      KREQ
mut_res                 38                      KREQ
mut_res                 42                      DKRQNSTEH
mut_res                 43                      DKRQNSTEH
mut_res                 49                      KREQ
mut_res                 56                      KREQ
mut_res                 60                      KREQ
mut_res                 64                      KREQ
mut_res                 75                      DKRQNSTEH
mut_res                 78                      DKRQNSTEH
mut_res                 79                      KREQ
mut_res                 82                      KREQ
mut_res                 85                      KREQ
mut_res                 88                      KREQ
mut_res                 89                      KREQ
mut_res                 111                     KREQ
mut_res                 112                     KREQ
mut_res                 118                     KREQ

# repacking residues
rpk_res                 17
rpk_res                 31
rpk_res                 35
rpk_res                 39
rpk_res                 53
rpk_res                 57
rpk_res                 67
rpk_res                 81
rpk_res                 92
rpk_res                 93
rpk_res                 96
rpk_res                 115
rpk_res                 119

# sampling parameters (optional)
scramble_order                  1 # default:= 0
m_mutations                     3 # default:= 3
n_paths                         7 # default:= 1
n_iters                         5 # default:= 1

# mutagenesis scoring weights (optional)
mut_max_lj                      25.0
mut_w_pp                        1.0
mut_w_k                         0.0
mut_w_lj                        1.0
mut_w_solv                      1.0
mut_w_elec                      0.125

# repacking scoring weights (optional)
rpk_max_lj              25.0
rpk_w_pp                1.0
rpk_w_k                 1.0
rpk_w_lj                1.0
rpk_w_solv              1.0
rpk_w_elec              0.125
```

Supplemental references

1. Vitalini, F., Noé, F., and Keller, B.G. (2016). Molecular dynamics simulations data of the twenty encoded amino acids in different force fields. Data Brief *7*, 582-590. 10.1016/j.dib.2016.02.086.
2. Shapovalov, M.V., and Dunbrack, R.L., Jr. (2011). A smoothed backbone-dependent rotamer library for proteins derived from adaptive kernel density estimates and regressions. Structure *19*, 844-858. 10.1016/j.str.2011.03.019.
3. Blaabjerg, L.M., Kassem, M.M., Good, L.L., Jonsson, N., Cagiada, M., Johansson, K.E., Boomsma, W., Stein, A., and Lindorff-Larsen, K. (2023). Rapid protein stability prediction using deep learning representations. Elife *12*. 10.7554/eLife.82593.
4. Rapaport, D.C. (2022). GPU molecular dynamics: Algorithms and performance. Journal of Physics: Conference Series *2241*, 012007. 10.1088/1742-6596/2241/1/012007.
